# Periodic Table Exploration of MXenes for Efficient Electrochemical Nitrate Reduction to Ammonia

**DOI:** 10.1002/smll.202410105

**Published:** 2025-02-19

**Authors:** Radhika Nittoor‐Veedu, Xiaohui Ju, Michal Langer, Wanli Gao, Michal Otyepka, Martin Pumera

**Affiliations:** ^1^ Quantum Materials Laboratory 3D Printing & Innovation Hub Center for Nanorobotics and Machine Intelligence Department of Chemical and Biochemistry Mendel University Zemědělská 1 Brno 61300 Czech Republic; ^2^ Future Energy and Innovation Laboratory Central European Institute of Technology Brno University of Technology Purkyňova 123 Brno 61200 Czech Republic; ^3^ IT4Innovations VSB – Technical University of Ostrava 17. listopadu 2172/15 Ostrava‐Poruba 70800 Czech Republic; ^4^ Czech Advanced Technology and Research Institute (CATRIN) Regional Centre of Advanced Technologies and Materials Palacký University Olomouc Šlechtitelů 27 Olomouc 77900 Czech Republic; ^5^ Department of Medical Research China Medical University Hospital China Medical University No. 91 Hsueh‐Shih Road Taichung 40402 Taiwan; ^6^ Department of Chemical and Biomolecular Engineering Yonsei University 50 Yonsei‐ro Seodaemun‐gu Seoul 03722 South Korea; ^7^ Energy Research Institute@NTU (ERI@N) Research Techno Plaza X‐Frontier Block Level 5 50 Nanyang Drive Singapore 637553 Singapore

**Keywords:** 2D materials, catalysis, electrochemistry

## Abstract

Applying electrochemical nitrate reduction reaction (NO_3_RR) to produce ammonia offers a sustainable alternative to the energy‐intensive Haber‐Bosch process, which is crucial for clean energy and agricultural applications. While 2D MXenes hold great promise as electrocatalysts for NO_3_RR, their application for ammonia production remains underexplored. This study combines experimental and theoretical approaches to evaluate the catalytic performance of a series of MXenes with different central metal atoms for NO_3_RR. Among the materials studied (Ti_3_C_2_T_x_, Ti_3_CNT_x_, Ti_2_CT_x_, V_2_CT_x_, Cr_2_CT_x_, Nb_2_CT_x_, and Ta_2_CT_x_), Ti_3_‐based MXenes exhibit superior faradaic efficiency, ammonia yield rate, and stability. Density functional theory calculations offer further insights explaining the structure‐activity‐based observations. This research establishes a foundation for future studies aimed at leveraging MXenes for electrochemical nitrate reduction for green synthesis of ammonia.

## Graphical abstract

Electrochemical nitrate reduction (NO_3_RR) offers a sustainable alternative to the Haber‐Bosch process for ammonia production. This study explores MXenes as electrocatalysts, analyzing their performance experimentally and theoretically. Ti_3_XT_x_ MXenes demonstrated superior efficiency, ammonia yield, and stability. Density functional theory elucidates structure‐activity relationships, paving the way for future MXene‐based research in green ammonia synthesis via electrochemical NO_3_RR.

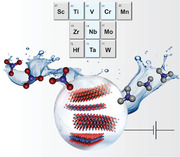

## 1 Introduction

Ammonia ranks as the second most produced chemical globally with its primary use in the agriculture sector.^[^ ^1^, ^2^, ^3^ ^]^ It should be noted that ammonia is also a viable option as hydrogen storage and transport media for hydrogen‐powered fuel cells due to its high energy density, high hydrogen content, and ease of liquefaction.^[^ ^4^, ^5^, ^6^ ^]^ However, the commercial production of ammonia still relies on a hundred years old Haber‐Bosch method, which is known for its harsh operating conditions. The extremely high temperature and pressure required for the optimum condition and the release of large amounts of CO_2_ to the environment make this method environmentally unpleasant. The electrochemical reduction method for ammonia synthesis provides a safe and environmentally friendly approach and presents as the best alternative for the current energy‐intensive Haber‐Bosch process.^[^ ^7^ ^]^ Along with ammonia synthesis, nitrate reduction is also known due to the concerning levels of nitrate pollution in groundwater, and the high costs associated with its effective removal from industrial and sewage wastewater.^[^ ^8^, ^9^ ^]^ To advance the commercialization of electrochemical nitrate reduction for ammonia synthesis, the development of efficient electrocatalysts is imperative. Such catalysts are essential to improving the selectivity, yield, and overall efficiency of the process, thereby enabling a more sustainable and practical approach to ammonia production.

MXenes belong to the emerging class of 2D transition metal carbides or nitrides with a general formula of M_n+1_X_n_T_x_, where M is the transition metal, X is carbon and/or nitrogen, and T is the surface functional groups such as –OH, –Cl, –F, etc. MXenes have unique physiochemical and electrochemical properties, showing great potential in various fields including energy storage, sensors, optoelectronics, as well as biomedical and environmental applications.^[^ ^10^, ^11^, ^12^, ^13^ ^]^ However, their application in ammonia production via electrochemical nitrate reduction reaction (NO_3_RR) remains relatively unexplored. The excellent electrical conductivity, hydrophilicity, large specific surface area, rich surface terminations, and superior mechanical stability in harsh conditions, creating an optimal interaction and easy transfer of electrons between the media, make MXenes good catalysts for electrochemical systems. Additionally, electronic structures, kinetic stability, surface charge, etc. of MXenes can be altered by easy change of surface terminations, potentionally enhancing the performance of MXene.^[^ ^14^ ^]^ Studies on functionalized or metal‐doped MXenes emphasize their potential as support materials for nitrate reduction; however, a comprehensive comparison across the wide range of MXenes for practical applications remains largely unexplored.^[^ ^15^, ^16^, ^17^ ^]^ While theoretical studies suggest their suitability as catalysts for nitrate reduction to ammonia, experimental validation remains limited.^[^ ^15^, ^18^, ^19^ ^]^ Despite their potential as electrocatalysts for ammonia synthesis, several challenges impede their practical implementation. One significant obstacle is achieving high selectivity for ammonia production over competing reactions, such as the hydrogen evolution reaction (HER) and the formation of undesired nitrogen byproducts. The intrinsic catalytic activity of MXenes toward HER often results in considerable current loss and diminished efficiency in nitrate reduction. Additionally, the surface chemistry of MXenes, characterized by terminal groups such as ─OH and ─F, can restrict nitrate adsorption and obstruct active sites, further complicating their application.^[^ ^20^, ^21^ ^]^

This study explores the potential of various MXenes as electrocatalysts for nitrate reduction to ammonia. We systematically explored the MXenes families and assessed how variations in transition metals and carbon/nitride substitution influence performances such as faradaic efficiency (FE), yield rate (YR), and catalyst stability. First, the pristine titanium‐based MXene Ti_3_C_2_T_x,_ their complementary carbonitride Ti_3_CNT_x_, and lower metal counterparts Ti_2_CT_x_ were compared, followed by changing the transition metal element to vanadium (V_2_CT_x_), chromium (Cr_2_CT_x_), niobium (Nb_2_CT_x_), and tantalum (Ta_2_CT_x_). Ti‐based MXenes stand out as promising candidates for electrochemical nitrate reduction, among all the studied materials. We further provided theoretical insights demonstrating the potential of the selected MXenes to function as efficient catalysts for ammonia production. And how the surface termination can affect the performance of the catalyst. These findings highlight the fascinating structure‐activity relations in MXenes, where seemingly minor variations in composition can lead to dramatic differences in their electrocatalytic behavior for ammonia production.

## 2 Results and Discussion

### 2.1 Physicochemical Characterization of MXenes

**Figure 1:**
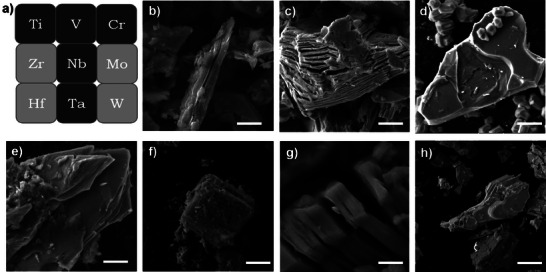
Morphological characterization of MXenes by scanning electron microscopy (SEM). a) Part of the periodic table including the transition metals of the selected MXenes for this study. SEM images of b) Ti_3_C_2_T_x_, c) Ti_3_CNT_x_, d) Ti_2_CT_x_, e) V_2_CT_x_, f) Cr_2_CT_x_, g) Nb_2_CT_x_, and h) Ta_2_CT_x_. The scale bars represent 2 µm.

All MXenes used in this study were purchased commercially and as‐received pristine materials were used without further treatment. First, comprehensive physicochemical characterization was carried out for all MXenes. **Figure** **1**a illustrates the positions of the transition metals of the MXenes used for the study on the periodic table, spanning across periods and groups. Preliminary morphological characterization by scanning electron microscopy (SEM) unveils a uniformly stacked layer structure across all samples including Ti_3_C_2_T_x_, Ti_3_CNT_x_, Ti_2_CT_x_, V_2_CT_x_, Cr_2_CT_x_, Nb_2_CT_x_, and Ta_2_CT_x_ (Figure 1b–h). These MXenes flakes exhibit size variations ranging from 0.2 to 4 µm, which is consistent with findings reported from previous studies.^[^ ^22^, ^23^, ^24^ ^]^

**Figure 2:**
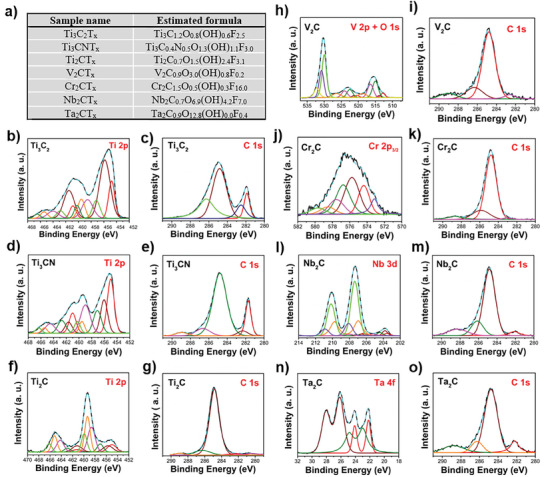
X‐ray photoelectron spectroscopy (XPS) measurement of as‐received MXenes. a) Table showing the estimated formula of MXenes. b–o) XPS core‐level spectra of b) Ti 2*p* of Ti_3_C_2_T_x_; c) C 1*s* of Ti_3_C_2_T_x_; d) Ti 2*p* of Ti_2_CNT_x_; e) C 1*s* of Ti_2_CNT_x_; f) Ti 2*p* of Ti_2_CT_x_; g) C 1*s* of Ti_2_CT_x_; h) V 2*p* + O 1*s* of V_2_CT_x_; i) C 1*s* of V_2_CT_x_; j) Cr 2*p_3/2_* of Cr_2_CT_x_; k) C 1*s* of Cr_2_CT_x_; l) Nb 3*d* of Nb_2_CT_x_; m) C 1*s* of Nb_2_CT_x_; n) Ta 4*f* of Ta_2_CT_x_; and o) C 1*s* of Ta_2_CT_x_. Fitting peak assignments and parameters are listed in Tables S1–S7 and Figures S1–S7 (Supporting Information).

Detailed characterization of these MXenes was carried out using X‐ray photoelectron spectroscopy (XPS) (**Figures** **2** and S1–S7, Supporting Information). XPS provides critical information on these MXenes to evaluate their chemical composition, oxidation states, and surface termination as received after extended exposure to the ambient environment. Figure 2a summarizes the calculated compositional formula resulting from XPS fitting, based on the proposed criteria by Halim et al.^[^ ^25^ ^]^ (detailed explanation in Note S1, Supporting Information). It needs to be noted that as complex as the structure is, these systems are challenging to characterize since their heterogeneity is due to the relatively large number of surface terminations and inner layered structure. XPS results of all MXenes confirm the M‐C (M = Ti, V, Cr, Nb, and Ta) bonding structure (Figure 2), although they are not always the dominant component due to extensive oxidation of the outer surfaces. The calculated M:X ratio is in good agreement with the structural ratio for all MXenes viewed from a stoichiometric perspective, remaining within the error range (±0.5). Some deficiency in X elements can be expected due to preferential HF etching of the X elements. For all samples the calculated –F_x_ termination is significantly higher than the reported values,^[^ ^25^ ^]^ which might be due to the intensive etching of the materials. For Ti_3_X_2_T_x_, replacing one C (Ti_3_C_2_T_x_) with N (Ti_3_CNT_x_) leads to an increase in the total amount of surface groups. In comparing Ti_3_X_2_T_x_ and Ti_2_XT_x_ MXenes, decreasing *n* leads to an increase in surface hydroxyl terminations, which is different from the previous report,^[^ ^25^ ^]^ where the hydroxyl moieties remain approximately the same. Most of the Ti species (Ti‐C, Ti‐C‐O/OH, C‐Ti‐F) belong to inner layered structures and surface terminations, while approximately a quarter of Ti species belong to the oxidized structure of TiO_x_/(‐F) (Figure 2b). The C 1*s* region of the as‐received Ti_3_C_2_T_x_ sample was fitted with 4 peaks (Figure 2c) with a combination of Gaussian and Lorentzian functions. The largest contribution comes from C‐C and C‐H(O). These contributions are likely due to the graphitic C‐C formation during the Ti dissolution of etching, in combination with factors like the remaining solvents used during the preparation process and sample exposure to the ambient atmosphere. C‐Al attributed to incomplete etching was also observed. Due to such contamination, the component related to the Ti‐C structure is observed only at a fraction ≈10%, as shown in Table S1 (Supporting Information). When replacing Ti with other transition metals, the surface oxygen termination dominates the functional groups for V_2_CT_x_, Ta_2_CT_x,_ and Nb_2_CT_x_, while Ta_2_CT_x_ possesses extremely high surface oxygen termination with negligible hydroxyl groups (Figure 2f–o). Further explanation of peak assignment and analysis of other elements such as O, F, and Al are elaborated in detail in Note S1 (Supporting Information). All fitted peak parameters are listed in Tables S1–S7 (Supporting Information) and core‐level spectra are shown in Figures S1–S7 (Supporting Information). To summarize, all characterized MXenes show corresponding M‐C structures (although limited by the probing depth of the XPS technique) and various degrees of surface termination groups. Another important point to note is that all analyzed MXenes show an extensive degree of metal oxide formation on their surfaces, which is inevitable due to the exposure to ambient conditions during material transportation and handling processes.

**Figure 3:**
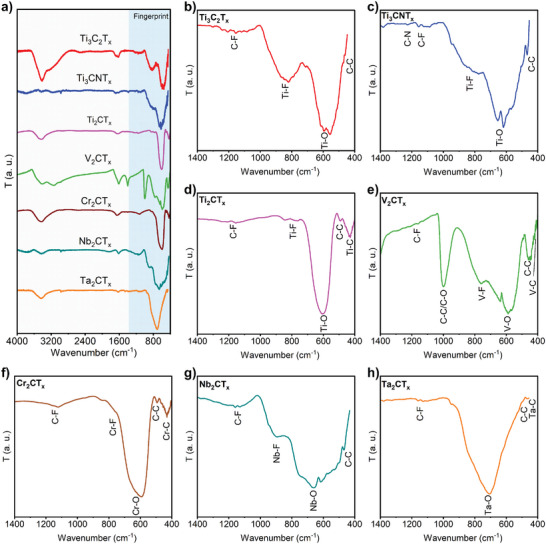
FTIR spectroscopy characterization of MXenes. a) FTIR spectra ranging from 4000–400 cm^−1^ of all as received MXenes and plotted after normalization (without smoothing). b–h) FTIR spectrum of individual MXenes in the fingerprint range of 1400–400 cm^−1^ with assigned bond vibrations. T stands for transmittance. b) Ti_3_C_2_T_x_; c) Ti_3_CNT_x_; d) Ti_2_CT_x_; e) V_2_CT_x_; f) Cr_2_CT_x_; g) Nb_2_CT_x_; and h) Ta_2_CT_x_.

The as‐received MXenes exhibit significant variability in their surface terminations, including ‐F, ═O, and ‐OH groups, arising from the synthesis methods employed. To complement XPS characterization, seven types of as‐received MXenes were further analyzed using Fourier transform infrared spectroscopy (FTIR). Due to the varied IR absorptivity and low IR transmittance of MXenes, the KBr pellet method was utilized for sample preparation and measurement. The resulting FTIR spectra, shown in **Figure** **3**a, reveal two major regions of interest. The 4000–1400 cm⁻¹ region includes several characteristic peaks: (1) ‐OH stretching and bending peaks at 3600–3200 cm⁻¹, attributed to confined water/moisture and ‐OH surface terminations, though differentiation is not possible at this stage; (2) peaks in the 3000–2800 cm⁻¹ range, assigned to C‐H stretching; and (3) peaks in the 1750–1400 cm⁻¹ range, attributed to C═O stretching, C‐O stretching, C‐H bending, and O‐H bending. The 1700–1550 cm⁻¹ region specifically corresponds to contributions from C‐O stretching and O‐H bending vibrations, primarily dominated by the C‐O stretching characteristic of the C‐T_x_ structure in MXenes (Figure 3b–h), as confirmed by previous studies.^[^ ^26^ ^]^ The 1400–400 cm⁻¹ region constitutes the fingerprint area for MXenes, with specific assignments including C‐F stretching (1400–1000 cm⁻¹), M‐F bending (750–700 cm⁻¹), M‐O bending (650–550 cm⁻¹), C‐C bending (500–400 cm⁻¹), and M‐C stretching (450–350 cm⁻¹). However, identifying surface terminations such as ‐OH and ═ O in FTIR is often challenging due to the dominant peaks from confined water and carbon bonds. Furthermore, the assignment of M‐F peaks is complicated by the complex coordination of MXenes structure, which frequently results in redshifts from the typical 1000–850 cm⁻¹ range to lower wavenumbers, as observed in Ti_3_C_2_T_x_.^[^ ^26^ ^]^ Despite these challenges, the FTIR spectra of as‐received MXenes show good agreement with XPS structural analysis, with most samples exhibiting ‐F and ‐O/ ═ O surface terminations. However, no ‐F termination was observed in the Ta_2_CT_x_ sample, which was instead dominated by its oxides (Ta‐O).

**Figure 4:**
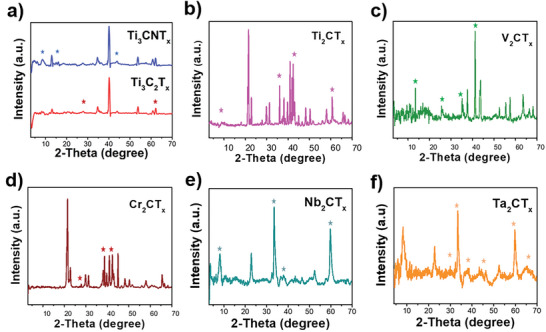
X‐ray diffraction (XRD) measurement of as‐received MXenes. a) Ti_3_C_2_T_x_ and Ti_3_CNT_x_; b) Ti_2_CT_x_; c) V_2_CT_x_; d) Cr_2_CT_x_; e) Nb_2_CT_x_; and f) Ta_2_CT_x_.

The X‐ray diffraction (XRD) analysis of the as‐received MXenes was carried out to investigate the crystallinity of all the MXene samples (**Figure** **4**). All the peaks marked with asterisks originate from the lattice planes of M_3_CT_x_/M_2_CT_x_. Figure 4a illustrates the XRD pattern of the pristine Ti_3_C_2_T_x_ and its nitride derivative Ti_3_CNT_x_. The peaks at 2θ values of 27.5° and 61.4° in the Ti_3_C_2_T_x_ pattern originate from the (008) and (110) planes, respectively, with other significant peaks corresponding to the unetched MAX phase.^[^ ^27^ ^]^ The substitution of one carbon atom with nitrogen in Ti_3_CNT_x_ does not substantially alter its crystal lattice, nonetheless, the strong peak at 8.5° corresponds to the (002) plane is revealed properly.^[^ ^28^ ^]^ The XRD pattern of lower coordination derivative Ti_2_CT_x_ is shown in Figure 4b with the marked asterisks for the 2θ value of 7.8°and 34.0° originates from the (002) and (101) crystal lattice planes, respectively, and the unetched Ti_2_AlC and TiC impurity contributes to the other major peaks.^[^ ^29^, ^30^, ^31^ ^]^ The V_2_CT_x_ peaks (Figure 4c) at 2θ values of 13.4°, 24.8°, and 41.2° align with the (004), (006), and (103) crystal phases of V_2_CT_x_ in its hexagonal phase as reported in the literature.^[^ ^32^ ^]^ Nevertheless, the other significant peaks observed at 37.2° and 43.0° may be attributed to VC impurities.^[^ ^33^ ^]^ The key peaks for Cr_2_CT_x_ lattice planes are observed at 27.9°, 37.4°, and 40.6° (Figure 4d) while the major peak at 21.0° and other observed peaks are attributed to Cr_3_C_7_ and other chromium carbide impurities respectively.^[^ ^34^, ^35^, ^36^, ^37^ ^]^ The diffraction from the lattice planes (002), (100), (101), and (110)^[^ ^38^, ^39^ ^]^ are observed at 9.0°, 33.7°, 38.0°, and 60.0°, respectively, for Nb_2_CT_x_ as shown in Figure 4e. The XRD pattern in Figure 4f with peaks observed at 31.7° (011), 33.1° (100), 37.3° (101), 45.4° (020), 60.0° (110), and 66.2° (122) confirms the presence of crystalline Ta_2_CT_x_.^[^ ^23^, ^40^ ^]^

### 2.2 Electrochemical Properties

**Figure 5:**
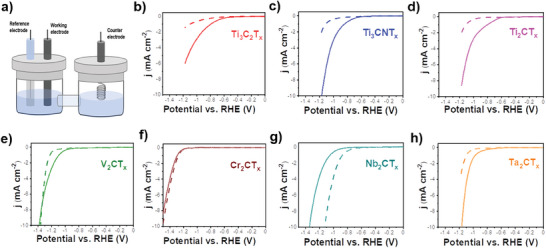
Electrocatalytic performance of all MXenes. a) electrochemical H‐cell setup; b–h) linear sweep voltammogram (LSV) in 0.5 m Na_2_SO_4_ (dashed line) and in 0.5 m Na_2_SO_4_+ 0.1 m KNO_3_ (solid line) with 5 mV s^−1^ scan rate of b) Ti_3_C_2_T_x_; c) Ti_3_CNT_x_; d) Ti_2_CT_x_; e) V_2_CT_x_; f) Cr_2_CT_x_; g) Nb_2_CT_x_; and h) Ta_2_CT_x_.

Regardless of whether the impurities in these as‐received MXenes originate from the production process (such as residual Al, F, and organic components) or from handling processes (introduction of a relative portion of passivated metal oxide layers),^[^ ^41^ ^]^ we further utilized these MXenes to explore their electrochemical performance for nitrate reduction for ammonia production. The linear sweep voltammetry (LSV) experiments were conducted in two different electrolytes: 0.5 m Na_2_SO_4_ and 0.5 m Na_2_SO_4_ + 0.1 m KNO_3_ solutions to determine the electrochemical nitrate reduction performance of the catalyst (**Figure** **5**). Figure 5a depicts the H‐cell setup for electrochemical nitrate reduction, with the cathodic and anodic chambers separated by a frit. These results unveil varying activities among the MXenes samples for NO_3_RR. Notably, the Ti‐based MXenes (Ti_3_C_2_T_x_, Ti_3_CNT_x_, and Ti_2_CT_x_) demonstrate a significant shift in reduction potential upon nitrate ions addition, suggesting their promising competence for electrochemical nitrate reduction. Pristine Ti_3_C_2_T_x_ shows a significant enhancement in onset potential (≈−0.6 V vs. reversible hydrogen electrode, RHE) in the presence of nitrate compared to nitrate‐free conditions (Figure 5b) with a 400 mV shift. Substituting one carbon atom with nitrogen in Ti_3_CNT_x_ maintains this 400 mV enhancement, with an onset potential shifting from −1.1 to −0.7 V vs. RHE (Figure 5c) upon nitrate ion addition. Similarly, Ti_2_CT_x_ with a lower metal atom coordination (*n* ═ 2) also exhibits a noticeable onset potential shift of ≈380 mV (Figure 5d). These results collectively underscore the exceptional catalytic performance of all tested Ti‐based MXenes for electrochemical nitrate reduction.

We further investigated the nitrate reduction activity of other MXenes by altering the central metal atom from Ti to a selected range of metals across the periodic table in their most reported formats. Vanadium (V), chromium (Cr), niobium (Nb) and tantalum (Ta) are transition metals situated close to titanium (Ti) in the periodic table. This proximity suggests that the crystal structures of their carbides may be alike, characterized by strong metallic and covalent bonding due to the *d*‐orbital overlap and carbon's contribution to the lattice stability. V_2_CT_x_, with vanadium having one atomic number higher than titanium in Ti_2_CT_x_, displays a modest 250 mV shift in onset potential (Figure 5e), indicating its capability for nitrate reduction, albeit to a lesser extent than that of Ti_2_CT_x_. Intriguingly, Cr_2_CT_x_, with chromium positioned adjacent to vanadium in the periodic table, exhibited no discernible activity for NO_3_RR, with reduction potentials nearly overlapping regardless of the presence of nitrate ions (Figure 5f). Although Nb belongs to the same group as Ti, Nb_2_CT_x_ displays a significantly more negative reduction potential in nitrate solution (Figure 5g), hindering its electrocatalytic performance for nitrate reduction. Ta which is in the same group as Nb and Ti having the highest atomic number, shows restrained nitrate reduction performance with ≈200 mV vs. RHE increase in the onset potential as shown in Figure 5h, demonstrating a reduced performance than Ti_2_CT_x_ but comparable to V_2_CT_x_. Although LSV measurements do not disclose comprehensive electrocatalytic behavior, they offer initial guidelines for assessing ammonia production capabilities, as detailed in subsequent sections.

### 2.3 Electrochemical Ammonia Generation

To validate the observations from the LSV and further investigate the catalytic efficiency and ammonia production yield from MXenes electrocatalysis, additional electrolysis was conducted within the selected potential range, encompassing the reduction potentials of each sample. For this purpose, chronoamperometry (CA) was carried out for one hour at the chosen potential. The electrolyte was collected after one hour and subjected to further analysis for ammonia concentration calculation using the indophenol blue colorimetric method.^[^ ^42^ ^]^ UV‐Vis absorption spectroscopy analysis reveal the presence of sharp peaks at 655 nm wavelength following appropriate dilution of the electrolyte indicative of the presence of ammonia (Figure S8, Supporting Information).^[^ ^42^ ^]^

**Figure 6:**
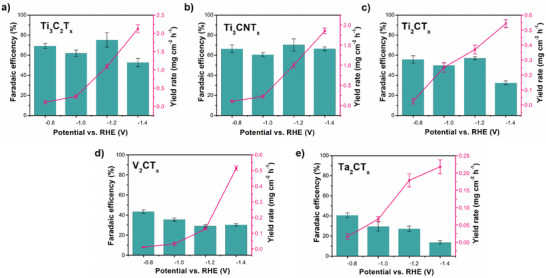
Faradaic efficiency (FE) and yield rate (YR) of ammonia production based on the electrochemical nitrate reduction catalyzed by a) Ti_3_C_2_T_x_; b) Ti_3_CNT_x_; c) Ti_2_CT_x_; d) V_2_CT_x_; and e) Ta_2_CT_x_.

The FE and ammonia yield were calculated using the ammonia concentration obtained from the colorimetric method using Equations (1) and (2). **Figure** **6** compares the FE and YR of all the samples. Ti_3_C_2_T_x_ achieved a maximum FE of 75% at −1.2 V vs RHE, and the ammonia yield was obtained up to 1.2 mg cm^−2^ h^−1^ at the corresponding potential (Figure 6a). Ti_3_CNT_x_ exhibited a similar FE compared to Ti_3_C_2_T_x_ (≈70%) at −1.2 V vs RHE and a YR of 1.20 mg cm^−2^ h^−1^ was observed confirming that the change of the presence of nitrides did not influence the performance to great extent (Figure 6b). However, reducing metal coordination to Ti_2_CT_x_ noticeably decreases the FE to 58% and ammonia yield by half compared to Ti_3_CNT_x_ (Figure 6c). As shown in Figure 6d,e, V_2_CT_x_ and Ta_2_CT_x_ exhibited maximum FEs of ≈30%, with a reduced ammonia yield by about a factor of ten compared to Ti_3_ MXenes. The FE and YR of Cr_2_CT_x_ and Nb_2_CT_x_ were also calculated, where Nb_2_CT_x_ has a FE of less than 10% and Cr_2_CT_x_ exhibits FE of less than 20% with a very low yield of ammonia (Figure S9, Supporting Information). The results correspond well with the trend observed by LSV, where Nb‐ and Cr‐based MXenes have the lowest electrocatalytic activity toward electrochemical nitrate reduction. The high FE observed at −0.8 V is likely due to the limitations of the colorimetric method in detecting very low ammonia yields, which can be significantly influenced by environmental contaminants.

**Figure 7:**
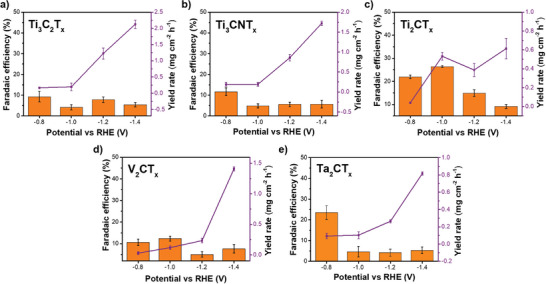
Faradaic efficiency (FE) and yield rate (YR) of nitrite production based on the electrochemical nitrate reduction catalyzed by a) Ti_3_C_2_T_x_; b) Ti_3_CNT_x_; c) Ti_2_CT_x_; d) V_2_CT_x_; and e) Ta_2_CT_x_.

To assess the selectivity of MXenes for ammonia synthesis, nitrite (NO_2_^−^) production was quantified using the Griess method, employing sulfanilamide and N‐(1‐naphthyl) ethylenediamine dihydrochloride (NED) as reagents. The characteristic absorption peak at 540 nm confirmed the presence of nitrite, and its concentration was determined using a calibration curve (Figure S12, Supporting Information). The resulting FE for nitrite (**Figure** **7**a–e) was significantly lower than those for ammonia, demonstrating the inherent selectivity of the MXenes toward ammonia production. Notably, the Ti₃‐based MXenes exhibited the lowest nitrite FEs among the materials tested, indicating their superior selectivity. However, despite the lower FEs, the nitrite yields were comparable to the ammonia yields across all five MXenes and even higher for V_2_CT_x_ and Ta_2_CT_x_, suggesting that further optimization is necessary to enhance selectivity and yield rate and achieve improved overall performance.

**Figure 8:**
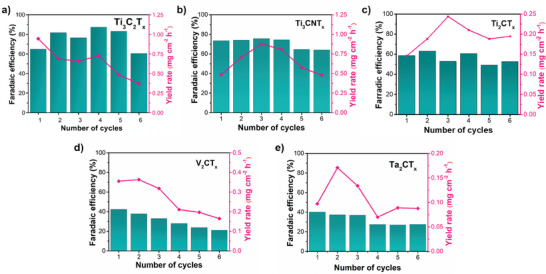
Cyclic stability of MXenes as electrocatalysts for nitrate reduction for ammonia production a) Ti_3_C_2_T_x_; b) Ti_3_CNT_x_; c) Ti_2_CT_x_; d) V_2_CT_x_; and e) Ta_2_CT_x_.

Following the determination of FE and YR, the stability of the selected MXenes for the electrochemical ammonia synthesis was evaluated for up to six cycles. **Figure** **8** summarizes the cyclic stability of all MXenes as electrocatalysts for NO_3_RR. Ti_3_C_2_T_x_ demonstrates consistent performance with an FE ranging from 65% to 85% across multiple cycles, although ammonia yield gradually decreased after each cycle (Figure 8a). Ti_3_CNT_x_ exhibits exceptional stability, maintaining a high FE for four cycles before a slight decline. While ammonia yield remained relatively constant ranging from 0.5 to 0.8 mg cm^−2^h^−1^, showcasing its promising potential as a stable catalyst (Figure 8b). Ti_2_CT_x_ displays comparatively stable FE and YR throughout each cycle (Figure 8c). V_2_CT_x_ shows lower initial FE compared to the Ti‐based MXenes, with a gradual decline from 40% to ≈20% over six cycles, along with following the same trend for ammonia yield as shown in Figure 8d. Despite the low FE, Ta_2_CT_x_ exhibits promising stability, preserving FE for the first three cycles and a slight decrease in FE of ≈10% occurred in the fourth cycle, after which the performance stabilized for the remaining cycles (Figure 8e).

**Figure 9:**
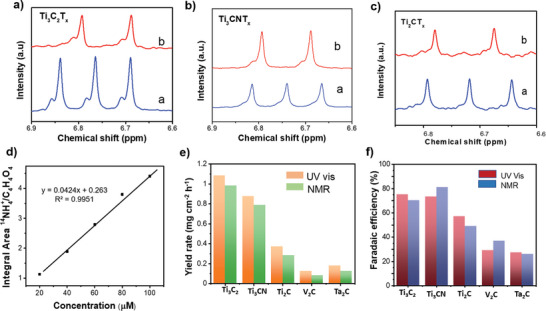
Nuclear magnetic resonance study of origin of NH_4_^+^. ^1^H NMR spectra of the electrolyte after electrocatalytic nitrate‐to‐ammonia conversion at −1.2 V, using K^14^NO_3_ (a) and K^15^NO_3_ (b) as the nitrogen source, catalyzed by a) Ti_3_C_2_T_x_, b) Ti_3_CNT_x_, c) Ti_2_CT_x_. d) Calibration curve for ammonia determination using the ^1^H NMR method. e) Comparison of yield rate for ammonia quantified using colorimetric indophenol blue method and _1_H NMR. f) Comparison of faradaic efficiency for ammonia quantified using colorimetric indophenol blue method and ^1^H NMR.

In order to confirm the origin of NH_4_^+^ in the electrolyte after 1 hr of electrolysis, 1H proton NMR studies were conducted using K^14^NO_3_ and K^15^NO_3_ as the nitrogen source as shown in **Figure** **9**. The distinctive doublet is observed for the electrolyte from K^15^NO_3_ as the source, and a triplet is observed for K^14^NO_3_ for all the MXene‐catalyzed electrolysis. The ^1^H NMR spectra for Ti‐based MXene are shown in Figure 9a–c with sharp intensity for the peaks corresponding to different isotopes of nitrogen. The V_2_CT_x_ and Ta_2_CT_x_ showed a very low intensive peak for both ^15^N and ^14^N showing their lower ammonia production compared to Ti‐based MXenes (Figure S14, Supporting Information). ^1^H NMR calibration curve was obtained using the known concentrations of ^14^NH_4_Cl using the maleic acid as an internal standard, shown in Figure 9d. The integrated area under the curve (Figure S13, Supporting Information) was calculated relative to the internal standard. The FE and YR obtained from the ^1^H NMR quantification was compared with results obtained using the indophenol method via UV–vis spectroscopy and both quantification methods produced a comparable value (Figure 9e,f).

### 2.4 Insight from Density Functional Theory Calculations

**Figure 10:**
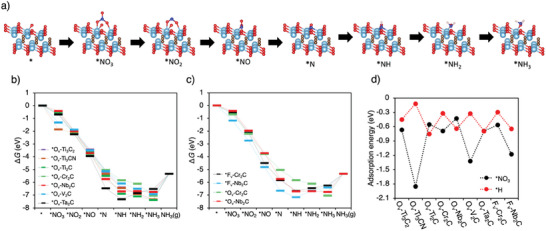
Theoretical study of selected MXenes for electrochemical NO_3_RR. a) DFT optimized structures of NO_3_RR intermediates adsorbed on O_v_‐Ti_2_C MXene. Coloring: Ti‐sky blue, O‐red, N‐dark blue, C‐brown, H‐white. Gibbs free energy diagram of NO_3_RR (U = 0 SV) on models of b) O_v_‐MXenes and c) F_v_‐MXenes of F_v_‐Cr_2_C and F_v_‐Nb_2_C (their O_v_‐ counterparts are also displayed for clear comparison). Gibbs free energy diagram of the HER (U = 0 V) on the models of O_v_‐ and F_v_‐MXenes. d) Comparison of adsorption Gibbs free energies of NO_3_^−^ and H^+^ on the models of O_v_‐ and F_v_‐MXenes.

The efficiency of Ti_3_C_2_T_x_, Ti_3_CNT_x_, Ti_2_CT_x_, V_2_CT_x_, Cr_2_CT_x_, Nb_2_CT_x_, and Ta_2_CT_x_ electrocatalysts was compared by calculating the thermodynamics of nitrate reduction to ammonia via Density Functional Theory (DFT) calculations. Based on the XPS data and previously published models,^[^ ^15^, ^18^ ^]^ structural models of MXenes (Figures S15–S22, Supporting Information) with the –O or –F functionalized surfaces and a single vacancy acting as a reactive active center (O_v_‐ and F_v_‐MXenes, respectively) were employed for DFT calculations. The Gibbs free energy diagrams of NO_3_RR intermediates were constructed along a reaction pathway involving nine protons and eight electrons, following the sequence (**Figure** **10**a): NO_3_^–^ → *NO_3_ → *NO_2_ → *NO → *N → *NH → *NH_2_ → *NH_3_ → NH_3_(g).^[^ ^15^ ^]^ For –O terminated MXenes (O_v_‐Ti_3_C_2_, O_v_‐Ti_3_CN, O_v_‐Ti_2_C, O_v_‐Cr_2_C, O_v_‐Nb_2_C, O_v_‐Ta_2_C, O_v_‐V_2_C), The Gibbs free energy changes (∆*G*) for all reaction steps along the reduction pathway are negative across most of the electrocatalysts, except for O_v_‐Nb_2_C and O_v_‐Ta_2_C (Figure 10a). For O_v_‐Nb_2_C, the *NH → *NH_2_ step is slightly endothermic, with a ∆G of 0.02 eV. The deoxygenation steps catalyzed by O_v_‐Ta_2_C are exothermic, however, the hydrogenation steps *NH → *NH_2_ and *NH_2_ → *NH_3_ are endothermic. These results suggest that the MXenes studied could generally serve as effective catalysts for the NO_3_RR. Although experimental data indicated a higher FE of Ti_3_‐based MXenes compared to Ti_2_C, the calculated ∆*G* diagram of NO_3_RR did not reveal a significant difference in performance between the ideal surfaces of O_v_‐Ti_3_C_2_ and O_v_‐Ti_2_C. Based on the XPS and XRD characterization, it is difficult to rule out the contribution of impurities on the final experimentally quantified electrocatalytic performances of the as‐received samples, which complicates the interpretation of the theoretical prediction. Nevertheless, in agreement with experimental observation, all examined MXenes exhibit favorable energy potential for effective NO_3_RR electrocatalysts.

Nonetheless, although experimental data indicated that Cr‐ and Nb‐based MXenes are not effective NO_3_RR electrocatalysts, thermodynamic calculations did not reveal any significant hindrance to the NO_3_RR reaction on idealized O_v_‐Cr_2_C and O_v_‐Nb_2_C MXenes. We further addressed this point by examining the role of surface functional groups in detail. XPS analysis indicates that Cr_2_C and Nb_2_C MXenes contain substantially higher levels of fluorine compared to other MXenes studied. This prompted further DFT calculations focused on the –F terminated MXenes, specifically F_v_‐Cr_2_C and F_v_‐Nb_2_C. These calculations demonstrated that the Gibbs free energy changes for the *NH → *NH_2_ and *NH_2_ → *NH_3_ steps are positive for F_v_‐Cr_2_C and F_v_‐Nb_2_C (Figure 10c), indicating that F‐terminated MXenes have more challenging hydrogenation steps compared to their corresponding O‐terminated counterparts. These endothermic hydrogenation steps hence make NO_3_RR less feasible for F‐terminated Cr_2_CT_x_ and Nb_2_CT_x_. The potential determining step (PDS) for F_v_‐Cr_2_C and F_v_‐Nb_2_C is identified as *NH → *NH_2_ with ∆*G* values of 0.22 and 0.51 eV, respectively. This suggests that MXenes surfaces with a high degree of fluorination may not be suitable catalysts for producing ammonia from nitrate.

**Figure 11:**
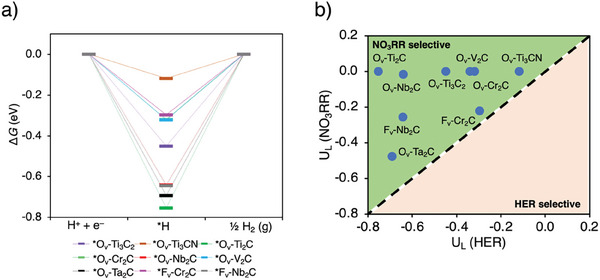
Theoretical study of selected MXenes for electrochemical HER: a) Gibbs free energy diagram of the HER (U = 0 V) on the models of O_v_‐ and F_v_‐MXenes; b) calculated limiting potentials for the HER (U_L_(HER), Volmer step) and NO_3_RR (U_L_(NO_3_RR)) on the surfaces of O_v_‐ and F_v_‐MXenes.

To better understand the competition between the NO_3_RR and HER, additional DFT calculations were conducted. The involvement of H^+^ in the NO_3_RR reaction introduces a competitive dynamic betweenHER and NO_3_RR, which significantly affects NO_3_RR selectivity.^[^ ^15^ ^]^ First, the adsorption Gibbs free energies of NO_3_^−^ ($\Delta G_{{*}_{NO_{3}}})$ and H^+^ ($\Delta G_{{*}_{H}}$) were compared (Figure 10d). The adsorption energies NO_3_^−^ are all negative for examined MXenes structures, ranging from −1.85 to −0.42 eV. This suggests that these materials could be considered good catalysts for NO_3_RR. Among them, O_v_‐Ti_3_CN MXene exhibits the strongest NO_3_^−^ adsorption to the MXene surface, followed by that of O_v_‐V_2_C. This can be attributed to their ability to stabilize the *NO_3_ intermediate by binding it to the MXenes surfaces with a single oxygen atom, even though initial binding before the DFT optimization was with two oxygen atoms of the NO_3_^−^molecule (Figures S15–S22, Supporting Information). In contrast, the calculated $\Delta G_{{*}_{H}}$ for the Volmer step of HER, *i.e*., the adsorption of the first hydrogen ranged from −0.75 to −0.12 eV (**Figure** **11**a). Notably, the $\Delta G_{{*}_{H}}$ for HER activity of O_v_‐Ti_2_C, O_v_‐Nb_2_C, and O_v_‐Ta_2_C show more negative values compared to $\Delta G_{{*}_{NO_{3}}}$, suggesting that these catalysts might favor HER over NO_3_RR based on this adsorption energy descriptor (Figure 10d). This could also contribute to explaination of the lower NO_3_RR activity of Ti_2_‐based MXenes vs. Ti_3_‐based MXenes. Nevertheless, when considering the limiting potential as a descriptor for NO_3_RR vs. HER competition,^[^ ^15^ ^]^ nitrate reduction to ammonia proves to be more favorable than HER. As shown in Figure 11b, the limiting potential for NO_3_RR [U_L_(NO_3_RR)] is less negative than for HER [U_L_(HER)], largely due to the exothermic nature of most NO_3_RR reaction steps. Therefore, based on the limiting potential as a descriptor, the MXenes we tested show greater selectivity toward NO_3_RR over HER in the Volmer–Heyrovsky pathway.

## 3 Conclusion

This study presents a significant advancement in the field of electrochemical nitrate reduction by rigorously evaluating the efficacy of the recently discovered 2D MXenes, thus highlighting their potential application in ammonia production. The experimental investigation in this study validates the previous theoretical prediction that MXenes possess the potential to be used for electrochemical nitrate‐to‐ammonia conversion. Various pristine MXenes from across the periodic table were utilized to corroborate the theoretical findings. Since the MXenes were used as‐received, their impurities and oxidation layers could potentially play a role in affecting their efficiency. Our initiation is to evaluate their potential possibility without further tunning detailed parameters such as oxidation states, surface termination groups, impurity effects, homogeneity, *etc*., thus these results bring significant importance for evaluating the potential application of MXenes for electrochemical ammonia production without complicated pre‐treatment processes.

The promisingly high faradaic efficiency and yield rate of Ti_3_C_2_T_x_ and Ti_3_CNT_x_ underscore the potential of these MXenes for efficient nitrate reduction to produce ammonia. However, the M_2_C derivatives from other transition metals across the periodic table exhibit significantly lower faradaic efficiency and ammonia yield rate, emphasizing the need for further investigation in this area to enhance their electrochemical performance.

DFT calculations on model MXenes with single vacancies reveal that O‐terminated MXenes are more favorable for nitrate reduction to ammonia, as all free energy changes are negative. In contrast, F‐terminated MXenes show positive free energy changes during certain hydrogenation steps, making them less efficient as catalysts. The impurities arise from the synthetic procedure and oxidation of MXenes affects their electrochemical properties to an extent. This study demonstrates the promise of Ti_3_C_2_T_x_ and Ti_3_CNT_x_ MXenes for electrocatalytic nitrate reduction reaction to ammonia. Inspired by these findings, further research efforts should focus on tuning the structure‐activity relationship to develop highly efficient and selective MXenes‐based electrocatalysts for applicable ammonia synthesis.

## 4 Experimental Section

### Materials

Ti_3_C_2_T_x_, Ti_3_CNT_x_, Ti_2_CT_x_, V_2_CT_x_, Nb_2_CT_x_, Cr_2_CT_x_, and Ta_2_CT_x_ were purchased from 2D semiconductors (USA). Ethanol (96%) was purchased from Penta Chemicals (Czech Republic). Nafion, sodium sulfate, sodium hydroxide, citric acid, sodium nitroferricyanide, *N*‐(1‐naphthyl) ethylenediamine dihydrochloride, sulfanilamide, phosphoric acid, sodium hypochlorite, maleic acid, Potassium nitrate (K^15^NO_3_)_,_ and salicylic acid were purchased from Merck (Germany). Potassium nitrate (K^14^NO_3_) was purchased from Alfa Aesar. All the chemicals were analytical grade and used as received. All the solutions were prepared in Millipore water with a resistivity of 18 MΩ cm.

### Physicochemical Characterizations

The morphology of MXenes was studied by SEM (TESCAN MIRA 3 XMU). Samples were mounted on the carbon tape and measured at 10 kV. The chemical compositional analysis was carried out by XPS (Kratos Analytical Axis Supra) equipped with a monochromatized Al K*α* X‐ray source (1486.6 eV). The as‐received MXene‐powered samples were pressed onto a double‐sided copper tape. The X‐ray beam has a slit spot size of 200 × 700 µm. Charge neutralization was provided through an electron flood source at ≈3.5 eV. The survey spectra were obtained with a pass energy of 80 eV and step size of 1 eV while the core‐level spectra of all elements (Ti 2*p*, V 2*p*, Ta 4*f*, Cr 2*p*, Nb 3*d*, Al 2*p*, F 1*s*, N 1*s*, O 1*s* and C 1*s*) were collected with a pass energy of 20 eV and step size of 0.1 eV. All spectra were calibrated based on adventitious carbon at 284.8 eV and fitted using KolXPD software (http://kolxpd.kolibrik.net). XRD was obtained by using an X‐ray diffractometer (Rigaku SmartLab 3 kW) by setting up Brag Brentano geometry using a Cu K*α* radiation source. FTIR was measured by Bruker Vertex V70 FTIR spectrometer in the transmittance mode ranging from 4000–400 cm^−1^, with 1 cm^−1^ resolution and a total of 32 scans, and repeated 3 times. Baseline correction was performed using Opus software with concave rubberband correction, 2 iterations, 4 baseline points, and excluding CO_2_ peaks. The data was smoothed using a 50‐point average. As‐received MXenes (10 mg) were grounded with pre‐dried KBr (200 mg). The mixtures were prepared into KBr pellets with a Pellet pressing die set with 13 mm in diameter at 60kN using a hydraulic press. Pure KBr was recorded as background. The origin of the ammonium ion was detected using a 1H proton NMR analysis with a 600 MHz NMR spectrometer (Bruker Avance NEO) and the data was analyzed using topspin software.

### Electrochemical Measurement

A potentiostat, (PGSTAT 204, Metrohm Autolab, Netherlands) connected to a computer and controlled by NOVA software (version 2.1) was used for all the voltammetry measurements. All the electrochemical measurements were performed using a three‐electrodes setup, with Pt as the counter electrode, Ag/AgCl as the reference electrode, and electrocatalyst‐coated glassy carbon as the working electrode. The MXenes were suspended in a 1:1 ethanol‐water mixture and 5 wt.% Nafion (4 mg.ml^−1^), followed by sonication in a bath sonicator for 20 minutes. The sonicated MXenes were coated on the glassy carbon electrode (30 µL) allowed to dry in the air and used as the working electrode.

The linear sweep voltammetry was measured in 0.5 M Na_2_SO_4_ solution and 0.5 M Na_2_SO_4_ + 0.1 M KNO_3_ with a scan rate of 5 mV s^−1^ to study the nitrate reduction activity. The electrolysis was performed in an H‐cell separated by a frit, in a 0.5 m Na_2_SO_4_ + 0.1 m KNO_3_ electrolyte, distributed evenly on both anodic and cathodic compartments (20 mL each). The electrolysis was performed for one hour for every MXene with different applied potentials with continuous stirring of 175 rpm to study the ammonia yield and FE. All the potential was converted into a reversible hydrogen electrode (RHE).

### Determination of Ammonia

The electrolytes after each electrolysis were collected and tested with a UV–vis spectrophotometer (JASCO V‐750) to quantify the ammonia production. A certain amount of electrolyte was collected and diluted up to 600 µL. A 600 µL of the following solution was added to the electrolyte, which contains 3 m NaOH, 10 wt.% salicylic acid, and 10 wt.% citric acid. Following the first solution, another solution (300 µL) containing 0.2 M NaClO and 2.0 wt.% sodium nitroferricyanide (60 µL) was added and the mixture rested for two hours. The concentration of ammonia was calculated using the formed indophenol blue product by measuring the absorbance at 655 nm wavelength and calculations based on the calibration curve using a series of standard NH_4_Cl solutions with 0.5 m Na_2_SO_4_.^[^ ^43^ ^]^ The FE and YR were calculated using the following equations:

(1) $$FE=\left(8\times F\times c_{NH_{3}}\times V\right)/Q$$

(2) $$YR=\left(C_{NH3}\times V\right)/\left(t\times s\right)$$

where *F* is the Faradaic constant (96 485 C mol^−1^), $c_{NH_{3}}$ is the concentration of measured NH_3_, V is the electrolyte volume of the cathodic compartment, *Q* is the total charge passing the electrode, *t* is the electrolysis time, and *S* is the surface area of the working electrode.

### Determination of Nitrite

The nitrite concentration in the electrolyte after electrolysis was determined using a method similar to that used for ammonia detection. The color reagent is prepared by combining the following components: N‐(1‐Naphthyl) ethylenediamine dihydrochloride (0.02 g), sulfanilamide (0.4 g), ultrapure water (5 mL), and phosphoric acid (1 mL, ρ  =  1.70 g/mL). The electrolyte sample was diluted to the appropriate concentration range, and 1.5 mL of the diluted sample was collected. To this, 50 µL of the color reagent was added, and the mixture was allowed to react for 20 minutes. The nitrite (NO₂⁻) concentration was determined by measuring the absorbance at a wavelength of 540 nm, using a calibration curve generated from a series of standard NaNO_2_ solutions. FE is calculated using the Equation (3) and the yield is calculated using the same equation used for ammonia quantification.

(3) $$FE=\left(2\times F\times c_{NH_{3}}\times V\right)/Q$$

### Computational Calculations

All DFT calculations were carried out using the Vienna Ab‐initio simulation package (VASP),^[^ ^44^, ^45^ ^]^ introducing the projector augmented wave (PAW)^[^ ^46^, ^47^ ^]^ potentials with a plane‐wave cutoff energy of 520 eV. The Perdew‐Burke‐Enrzerhof (PBE)^[^ ^48^ ^]^ parametrization of the generalized gradient approximation (GGA) was used to describe the electronic exchange‐correlation energies, and the DFT‐D3 method of Grimme with zero‐damping^[^ ^49^ ^]^ was also introduced to describe the van der Waals interactions. All structures were fully optimized until forces acting on all atoms were reduced to 0.03 eV Å^−1^, and electronic degrees of freedom were relaxed until the change in total electronic energy between successive iteration steps was smaller than 10^−5^ eV. The solvation effects of water were considered by VASPsol.^[^ ^50^ ^]^

The 3 × 3 × 1 supercell was used to model MXenes. The surface of these MXenes was fully covered with –O or –F atoms, and a single vacancy was introduced in each model. To eliminate the effects between two adjacent layers, a vacuum region of 15 Å was added in the z‐direction. For structural optimizations, the Brillouin zones integrations were performed with a gamma‐centered 3 × 3 × 1 Monkhorst‐Pack k‐point mesh grid.^[^ ^51^ ^]^

The computational hydrogen electrode (CHE)^[^ ^52^ ^]^ model was used to calculate the Gibbs free energy of reactions involving electron‐proton transfer; the temperature of 298.15 K was employed during the frequency calculations and VASPKIT^[^ ^53^ ^]^ was used for the post‐processing. The Gibbs free energy for nitrate NO_3_^−^ to adsorb on the MXene surface in aqueous solution forming *NO_3_ was calculated as:^[^ ^54^, ^55^ ^]^

(4) $$\;\Delta G_{{*}_{NO_{3}}}=G_{{*}_{NO_{3}}}-G_{*}-G_{{*}_{HNO_{3}\left(g\right)}}+\frac{1}{2}G_{H_{2}\left(g\right)}+0.392,$$

where $G_{{*}_{NO_{3}}}$, G_*_, $G_{{*}_{HNO_{3}\left(g\right)}}$, $G_{H_{2}\left(g\right)}$, and *G_correct_* are the Gibbs free energy of NO_3_^−^ adsorbed on O_v_/F_v_‐MXenes, O_v_/F_v_‐MXenes substrates, HNO_3_, and H_2_ molecules in the gas phase. The value 0.392 is the correction of adsorption energy.^[^ ^54^ ^]^

The Gibbs free energy of hydrogen adsorption (∆*G*_H_) at 0 V vs RHE was calculated as:

(5) $$\;\Delta G_{{*}_{H}}=G_{{*}_{H}}-G_{*}-\frac{1}{2}G_{H_{2}\left(g\right)}$$

Limiting potential^[^ ^56^ ^]^ *U*_L_ (*U*_L_ = ‐∆*G*_max_/e) was employed to describe the lowest bias requirement for the NO_3_RR and HER processes. Here, since NH_3_ can be removed by stirring and heating in experimental ways, the desorption of NH_3_ was not considered during the discussion of PDS.^[^ ^56^ ^]^

## Conflict of Interest

The authors declare no conflict of interest.

## Author Contributions

R.N.V. performed all the electrochemical measurements, SEM characterization, XRD analysis, and UV–vis spectroscopy measurements. X.J. performed the XPS measurement, analyzed the data, and revised the manuscript. M.L. performed theoretical calculations, analysis, and theoretical part writing. M.O. performed validation and revision of the theoretical part. M.P. provided the direction and supervised the project. W.G. supervised the experimental procedures for ammonia determination. All authors contributed to the manuscript.

## Supporting information



Supporting Information

## Data Availability

The data that support the findings of this study are available from the corresponding author upon reasonable request.
