# Supplementary material for: Periodic Table Exploration of MXenes for Efficient Electrochemical Nitrate Reduction to Ammonia
Source: Small. 2025 Feb 19;21(10):2410105. doi: 10.1002/smll.202410105 (PMC11899535; doi:10.1002/smll.202410105)
Supplement: Supplementary file 1 — Supporting Information [file SMLL-21-2410105-s001.docx]

Periodic Table Exploration of MXenes for Efficient Electrochemical Nitrate Reduction to Ammonia

*Radhika Nittoor-Veedu,^a^ Xiaohui Ju ^b^ Michal Langer,^e^ Wanli Gao^b^ Michal Otyepka,^e,f^ Martin Pumera^a,b,c,d,g*^*

^a^ Quantum Materials Laboratory, 3D Printing & Innovation Hub, Center for Nanorobotics and Machine Intelligence, Department of Chemical and Biochemistry, Mendel University, Zemědělská 1, Brno, 61300, Czech Republic

^b^ Future Energy and Innovation Laboratory, Central European Institute of Technology, Brno University of Technology, Purkyňova 123, Brno, 61200, Czech Republic

^c^ Department of Medical Research, China Medical University Hospital, China Medical University, No. 91 Hsueh-Shih Road, Taichung, 40402, Taiwan

^d^ Department of Chemical and Biomolecular Engineering, Yonsei University, 50 Yonsei-ro, Seodaemun-gu, Seoul, 03722, Korea

^e^ IT4Innovations, VSB – Technical University of Ostrava, 17. listopadu 2172/15, Ostrava-Poruba 70800, Czech Republic

^f^ Czech Advanced Technology and Research Institute (CATRIN), Regional Centre of Advanced Technologies and Materials, Palacký University, Šlechtitelů 27, Olomouc, 77900, Czech Republic

^g^ Energy Research Institute@NTU (ERI@N), Research Techno Plaza, X-Frontier Block, Level 5, 50 Nanyang Drive, 637553 Singapore, Singapore

*Author for correspondence: M. Pumera; [pumera.research@gmail.com](mailto:pumera.research@gmail.com)

**Supplemental Note S1: XPS peak fitting and analysis of MXenes**

All as-received MXene samples were analyzed by XPS to better understand their structure, surface terminations, as well as their oxidation extent after being stored in the air for an extended length of time. As complex as the structure is, these systems are challenging to characterize since their heterogeneity is due to the relatively large number of surface terminations and inner layered structure. The metal (M) atoms that are bonded to C atoms alone come from the central layers of the n>1 MXene flakes, usually attributing to the M-C peaks. Surface terminations –T_x_ where –T_x_ can be –O, –OH, –F_x_, and even –OH-H_2_O_ads_ can be sometimes separated from their main M-C peaks. It is also possible to identify M atoms that are bonded to O atoms in their oxide form (further possibly bonded to F atoms), as separated oxyfluoride species. It should be kept in mind that the sheer chemical complexity of MXene systems (from internal moieties and adventitious organic species to oxidation products) can introduce ambiguity in the spectra. The existence of numerous chemical species requires the inclusion of multiple fitting components in XPS analysis to accurately account for their presence. It is also important to note that some binary carbide impurity MAX phases may be present in the initial powders.

**Figure S1** shows the survey and core-level spectra of as-received Ti_3_C_2_T_x_ together with their fitted peaks (Ti 2*p* and C 1*s* displayed in **Fig. 2b**). The peak positions obtained from the fits are summarized in **Table S1**. From the Ti 2*p* region, most of the species (77%) are Ti atoms (Ti-C, Ti^2+^, Ti^3+^, C-Ti-F) that belong to the inner layer structure and surface terminations. This corresponds well to the reported value of the as-received sample with approximately a quarter of the Ti 2*p* region belonging to the oxidized structure of TiO_x_/(-F). The C 1*s* region of the as-received Ti_3_C_2_T_x_ sample was fitted with 4 peaks. The largest contribution comes from C-C and C-H(O). This contribution may result from the formation of graphitic C-C bonds during the Ti dissolution during etching, along with the solvents used in the preparation process and exposure to the ambient atmosphere. C-Al was also observed, which can be attributed to an incomplete etching. Due to such contamination, the component related to the Ti-C structure is observed at only a fraction of around 10%. The O 1*s* were fitted by 5 components, 32% attributed to surface terminations of C-Ti-O_x_ and C-Ti-(OH)_x_, 22% to oxidized TiO_x_ species, and 46% to Al_2_O_3_ and Al(OF)_x_ residues. A large contribution to this region is from organic contamination that overlaps with many other peaks in this region that are not possible to resolve. It should be noted that quantitative information should not be relied on from the O 1*s*. The major component of the F 1*s* region for the Ti_3_C_2_T_x_ was C-Ti-F_x_ and the byproduct of AlF_x_/Al(OF)_x_ during synthesis. The byproducts related to Al species were also confirmed by the core-level spectra in the Al 2*p* region. The spin-orbit split for Al 2*p_3/2_* and Al 2*p_1/2_* was not resolved due to the high signal-to-noise ratio and slanted background rising on the high-binding energy side. Based on the summarized results, the overall formulas for the as-received Ti_3_C_2_T_x_ composition are determined to be Ti_3_C_1.2_O_0.8_(OH)_0.6_F_2.5_. It is calculated without including TiO_x_ and other known oxide fractions. It is noted that the ratio of Ti and C resonates well with the structure of Ti_3_C_2_T_x_, and corresponds well with the experimentally determined values from Halim et al.^1^ If one assumes the charge of –O is –2, –F and –OH is –1, and Ti to be +4, the average C oxidation state in T_3_C_2_T_x_ can be calculated to be approximately –2.8,. Note that in Halim’s paper,^1^ they assumed the Ti valence to be comparable to those of TiO based on XANES measurement around 2.4. It follows that the net charge on an MX block is positive and is neutralized by the adsorption of various negative terminations. Our results exhibited a significantly higher value of surface termination contribution (F at 2.5 vs reported between 1.2 to 0.25). The high contribution of F is suspected to be a mixture of the dominant surface termination on this sample to be –F, via C-Ti-F_x_, in combination with surface residual byproducts generated from the HF washing. However, both theoretical and experimental results show the -F groups can be exchanged upon aging in ambient atmosphere since -OH termination is more favored energetically. On the other hand, the observation that Ti-MXenes have OH surface termination is interesting. TiC is known to react with water, dissociating the molecule into oxo groups. For the rest of MXenes, similar analysis and peak fitting were carried out, and detailed peak fitting results were shown ranging from **Table S1-S7** and **Fig. S1-S7**.

| **Region** | **Assigned to** | **BE (eV)** | **FWHM (eV)** | **Fraction** |
| --- | --- | --- | --- | --- |
| Ti 2p_3/2_ | Ti-C | 454.8 (6.1) | 1.1 | 0.16 |
|  | Ti^2+^-C-O/OH(-H_2_O_ads_) | 445.8 (5.6) | 2.0 | 0.47 |
|  | Ti^3+^-C-O/OH(-H_2_O_ads_) | 457.2 (5.7) | 1.4 | 0.10 |
|  | TiO_x_ | 458.6 (5.4) | 2.0 | 0.14 |
|  | Ti-O_2-x_-F_x_ | 459.5 (5.9) | 1.3 | 0.09 |
|  | C-Ti-F_x_ | 460.2 (6.3) | 1.1 | 0.04 |
| C 1s | C-Ti-T_x_ | 281.9 | 0.7 | 0.11 |
|  | C-Al | 282.5 | 1.2 | 0.10 |
|  | C-C | 284.8 | 1.8 | 0.51 |
|  | C-H/O | 286.3 | 2.0 | 0.28 |
| O 1s | TiO_x_ | 530.0 | 1.4 | 0.22 |
|  | C-Ti-O_x_ | 531.1 | 1.2 | 0.18 |
|  | C-Ti-(OH­)_x_ | 532.0 | 1.1 | 0.14 |
|  | Organic O, H_2_O_ads_ | 533.0 | 2.0 | 0.33 |
|  | Al(OF)_x_ | 534.7 | 1.9 | 0.13 |
| F 1s | C-Ti-F_x_ | 685.0 | 1.6 | 0.43 |
|  | Al-F_x_ | 686.3 | 1.8 | 0.37 |
|  | Al(OF)_x_ | 687.5 | 2.0 | 0.20 |
| Al 2p | Al_2_O­_3_ | 74.1 | 3.0 | 0.26 |
|  | Al-F_x_ | 75.9 | 2.0 | 0.68 |
|  | Al(OF)_x_ | 77.6 | 1.5 | 0.06 |

**Table S1.** Ti_3_C_2_T_x_ XPS peak assignment and peak fitting parameters. The numbers in brackets in the column are the spin-orbit splitting values of Ti 2*p_1/2_* and 2*p_3/2_*.


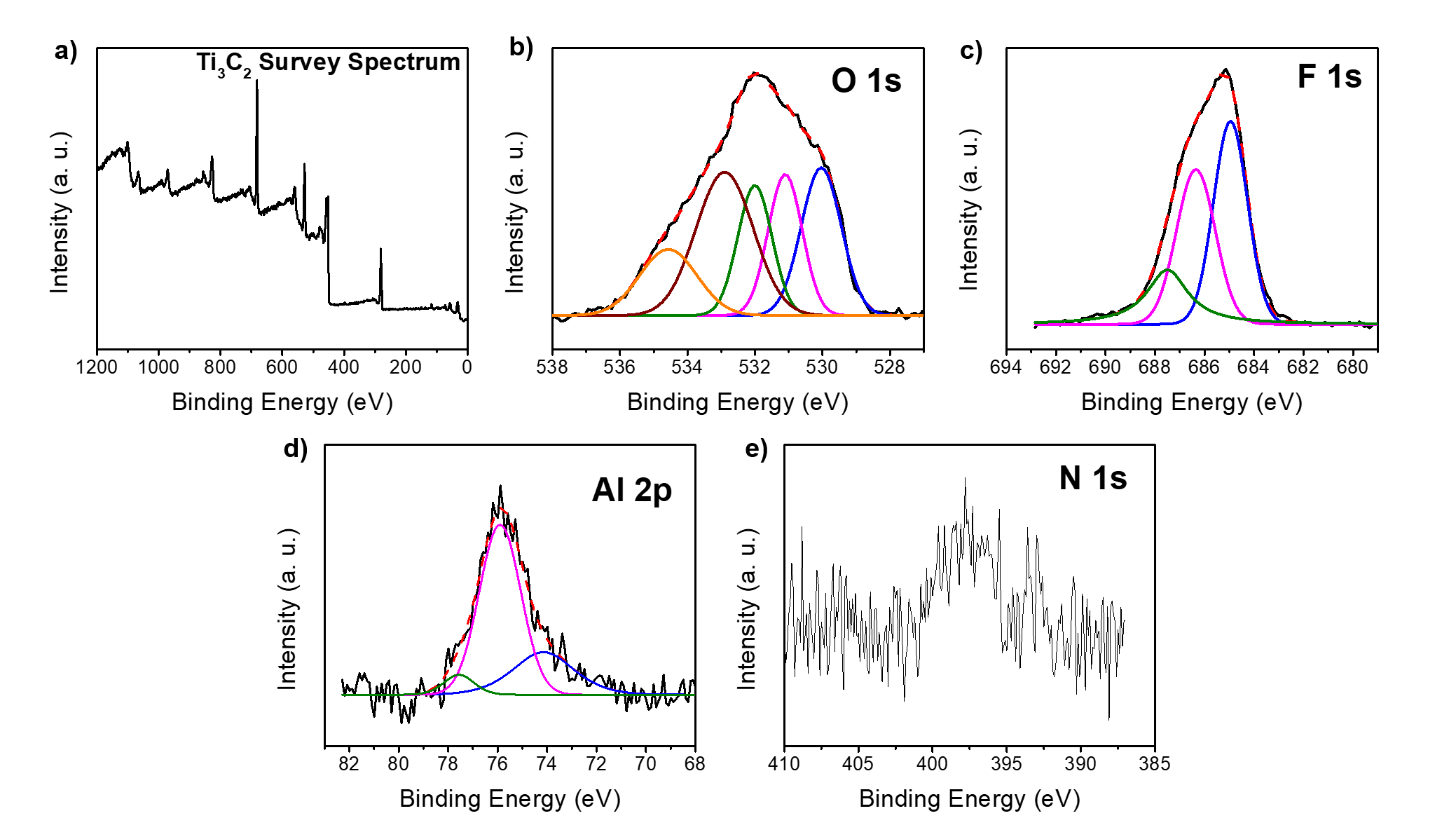


**Fig. S1**. **a)** Ti_3_C_2_T_x_ survey spectrum and core level spectra of **b)** O 1*s*, **c)** F 1*s*, **d)** Al 2*p*, and **e)** N 1*s.*

| **Region** | **Assigned to** | **BE (eV)** | **FWHM (eV)** | **Fraction** |
| --- | --- | --- | --- | --- |
| Ti 2p_3/2_ | Ti-C | 455.0 (6.0) | 1.3 | 0.31 |
|  | Ti^2+^-C-O/OH(-H_2_O_ads_) | 456.0 (5.6) | 1.3 | 0.19 |
|  | Ti^3+^-C-O/OH(-H_2_O_ads_) | 457.1 (5.6) | 1.5 | 0.17 |
|  | TiO_x_ | 459.0 (5.6) | 1.8 | 0.21 |
|  | Ti-O_2-x_-F_x_ | 459.5 (5.9) | 1.0 | 0.06 |
|  | NC-Ti-F_x_ | 460.2 (6.1) | 1.1 | 0.06 |
| C 1s | Ti-CN | 281.7 | 0.7 | 0.16 |
|  | NC-Ti-T_x_ | 282.3 | 1.0 | 0.04 |
|  | C-C | 284.8 | 1.8 | 0.71 |
|  | C-H/O | 286.7 | 1.8 | 0.07 |
|  | COO | 290.0 | 1.5 | 0.02 |
| N 1s | TiCN | 396.2 | 1.0 | 0.17 |
|  | NC-Ti-T_x_ | 397.2 | 0.9 | 0.30 |
|  | CH_3_CN | 399.3 | 2.0 | 0.13 |
|  | NH_4_NO_3_ | 402.0 | 2.5 | 0.40 |
| O 1s | TiO_x_ | 530.1 | 1.5 | 0.32 |
|  | NC-Ti-O_x_ | 531.1 | 1.3 | 0.22 |
|  | NC-Ti-(OH­)_x_ | 532.0 | 1.2 | 0.18 |
|  | Organic O, H_2_O_ads_ | 533.0 | 2.0 | 0.23 |
|  | Al(OF)_x_ | 534.7 | 1.6 | 0.05 |
| F 1s | NC-Ti-F_x_ | 684.9 | 1.7 | 0.55 |
|  | Al-F_x_ | 686.4 | 2.0 | 0.33 |
|  | Al(OF)_x_ | 687.1 | 2.0 | 0.07 |
|  | Organic F | 689.4 | 2.2 | 0.05 |
| Al 2p | Al-Ti | 72.2 | 1.0 | 0.12 |
|  | Al_2_O­_3_ | 74.0 | 2.0 | 0.06 |
|  | Al-F_x_ | 75.8 | 2.3 | 0.81 |
|  | Al(OF)_x_ | 77.9 | 1.0 | 0.01 |

**Table S2.** Ti_3_CNT_x_ XPS peak assignment and peak fitting parameters. The numbers in brackets in the column are the spin-orbit splitting values of Ti 2*p_1/2_* and Ti 2*p_3/2_*.


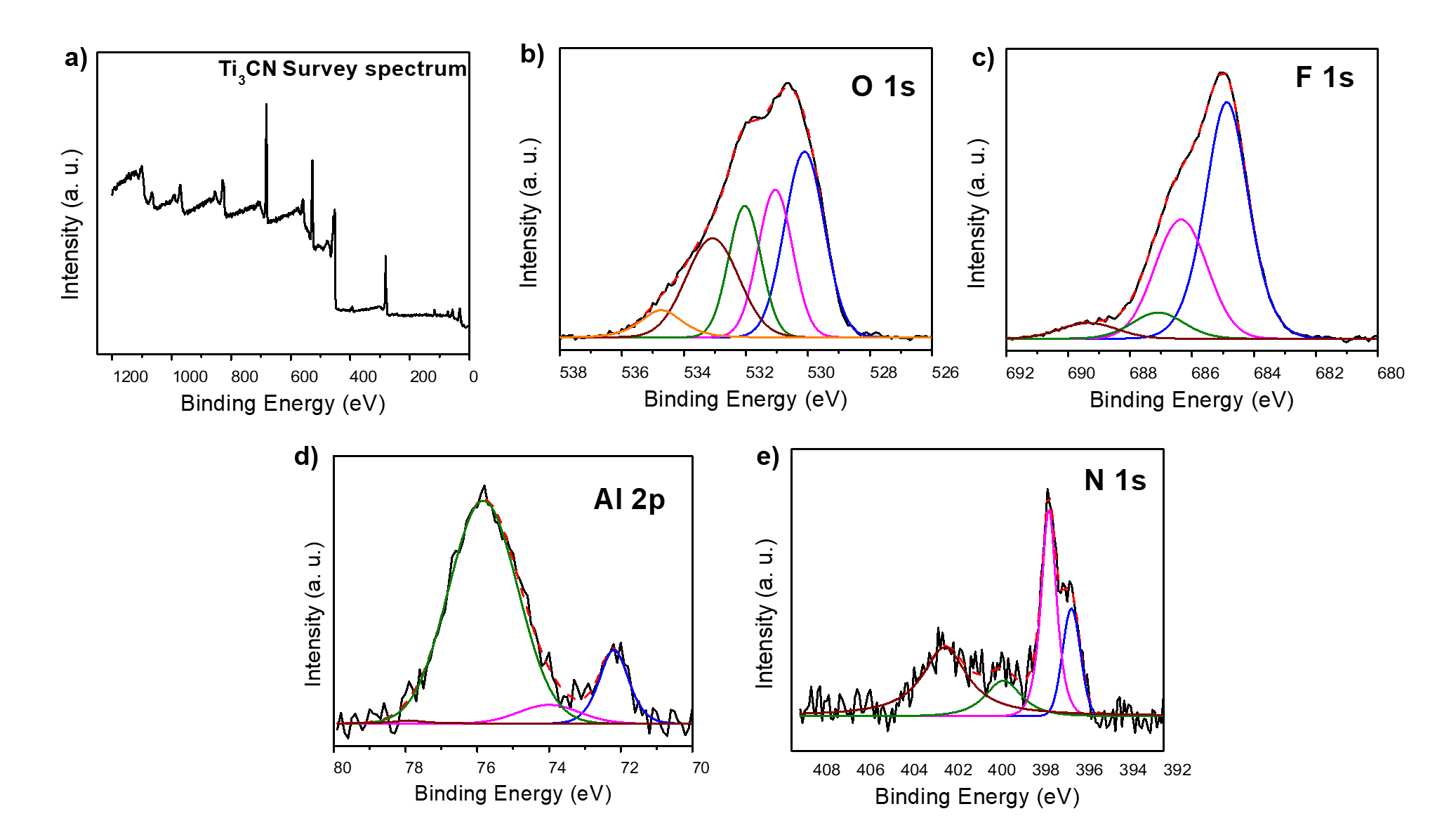


**Fig. S2.** **a)** Ti_3_CNT_x_ survey spectrum and core level spectra of **b)** O 1*s*, **c)** F 1*s*, **d)** Al 2*p*, and **e)** N 1*s*.

| **Region** | **Assigned to** | **BE (eV)** | **FWHM (eV)** | **Fraction** |
| --- | --- | --- | --- | --- |
| Ti 2p_3/2_ | Ti-C | 455.0 (6.2) | 1.9 | 0.09 |
|  | Ti^2+^-C-O/OH(-H_2_O_ads_) | 445.7 (5.5) | 2.0 | 0.12 |
|  | Ti^3+^-C-O/OH(-H_2_O_ads_) | 457.1 (5.8) | 1.3 | 0.07 |
|  | TiO_x_ | 458.6 (5.6) | 1.5 | 0.28 |
|  | Ti-O_2-x_-F_x_ | 459.4 (5.9) | 1.1 | 0.29 |
|  | C-Ti-F_x_ | 460.2 (6.3) | 1.1 | 0.15 |
| C 1s | HF-C | 280.5 | 1.6 | 0.03 |
|  | C-Ti-T_x_ | 282.3 | 2.0 | 0.01 |
|  | C-C | 284.8 | 1.4 | 0.83 |
|  | C-H/O | 286.3 | 2.2 | 0.09 |
|  | COO | 288.9 | 1.4 | 0.04 |
| O 1s | TiO_x_ | 530.0 | 1.8 | 0.41 |
|  | C-Ti-O_x_ | 531.1 | 1.3 | 0.07 |
|  | C-Ti-(OH­)_x_ | 532.0 | 1.0 | 0.11 |
|  | Organic O, H_2_O_ads_ | 533.0 | 1.7 | 0.27 |
|  | Al(OF)_x_ | 534.7 | 1.9 | 0.13 |
| F 1s | C-Ti-F_x_ | 685.0 | 2.0 | 0.02 |
|  | Al-F_x_ | 686.2 | 1.6 | 0.89 |
|  | Al(OF)_x_ | 687.5 | 2.0 | 0.09 |
| Al 2p | Al_2_O­_3_ | 74.0 | 1.6 | 0.04 |
|  | Al-F_x_ | 75.9 | 1.4 | 0.95 |
|  | Al(OF)_x_ | 77.6 | 1.9 | 0.01 |

**Table S3.** Ti_2_CT_x_ XPS peak assignment and peak fitting parameters. The numbers in brackets in the column are the spin-orbit splitting values of Ti 2*p_1/2_* and Ti 2*p_3/2_*.


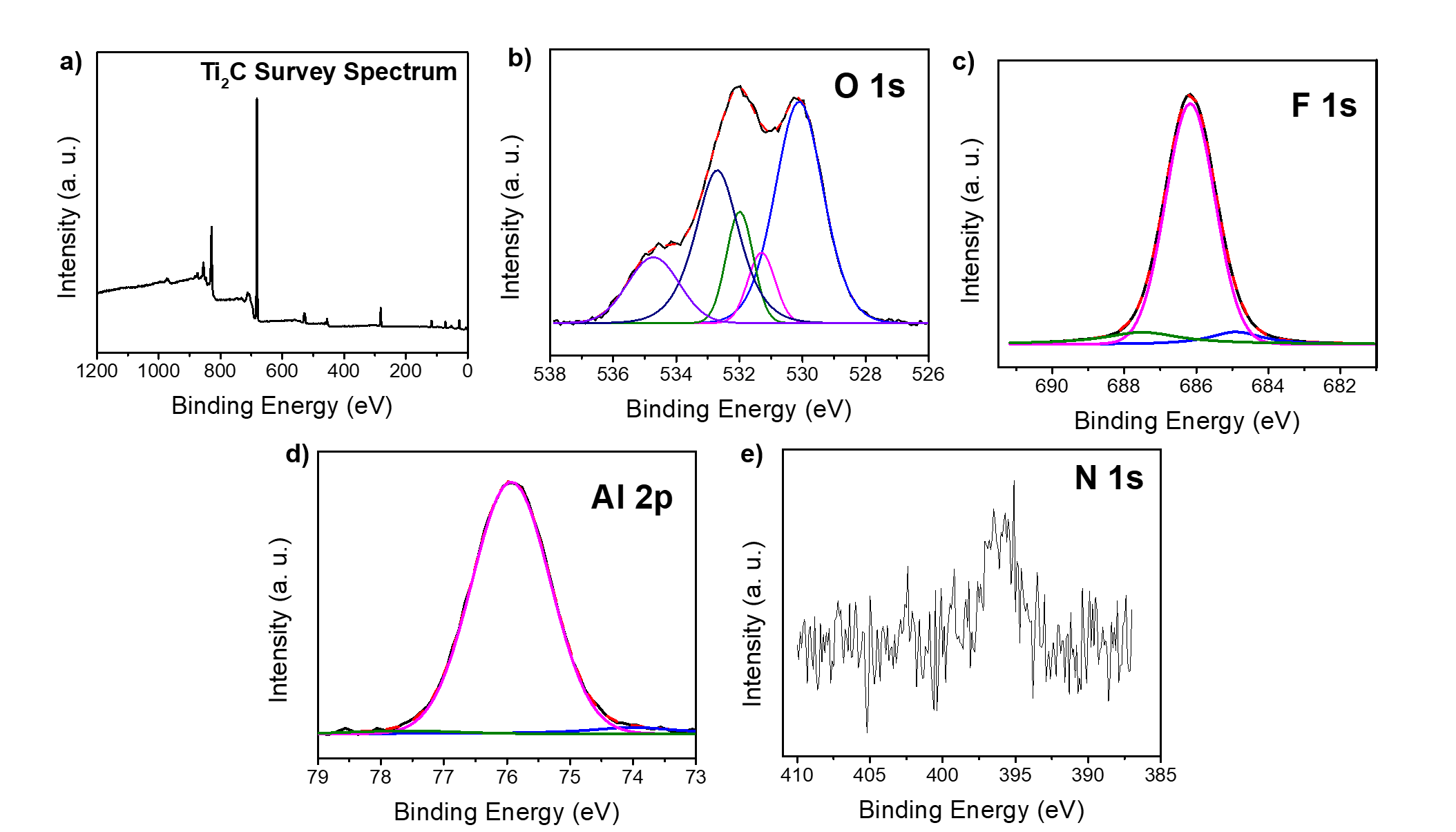


**Fig. S3. a)** Ti_2_CT_x_ survey spectrum and core level spectra of **b)** O 1*s*, **c)** F 1*s*, **d)** Al 2*p*, and **e)** N 1*s*.

| **Region** | **Assigned to** | **BE (eV)** | **FWHM (eV)** | **Fraction** |
| --- | --- | --- | --- | --- |
| V 2p_3/2_ | V-C | 512.8 (7.6) | 1.5 | 0.10 |
|  | V-C-O/OH(-H_2_O_ads_) | 513.9 (7.5) | 1.1 | 0.04 |
|  | V(3+)-O | 515.0 (7.5) | 1.9 | 0.37 |
|  | V(4+)-O | 516.4 (7.6) | 2.0 | 0.33 |
|  | V(5+)-O | 518.6 (7.6) | 2.5 | 0.16 |
| C 1s | C-Ti-T_x_ | 282.6 | 1.6 | 0.04 |
|  | C-C | 284.8 | 2.0 | 0.73 |
|  | C-H/O | 286.3 | 2.0 | 0.16 |
|  | C-OOH | 289.0 | 1.2 | 0.07 |
| O 1s | V-O | 530.0 | 1.3 | 0.30 |
|  | C-V-O_x_ | 530.8 | 2.0 | 0.68 |
|  | C-V-(OH­)_x_ | 532.2 | 1.2 | 0.02 |
| F 1s | C-Ti-F_x_ | 685.2 | 1.7 | 0.56 |
|  | Al-F_x_ | 686.4 | 1.2 | 0.26 |
|  | Al(OF)_x_ | 687.3 | 1.3 | 0.18 |
| Al 2p | Br contamination | 68.5 | 3.4 | Not counted |
|  | Al-Ti | 71.2 | 3.0 | 0.15 |
|  | Al-O­ | 74.6 | 1.5 | 0.78 |
|  | Al(OF)_x_ | 76.2 | 1.5 | 0.07 |

**Table S4.** V_2_CT_x_ XPS peak assignment and peak fitting parameters. The numbers in brackets in the column are the spin-orbit splitting values of V 2*p_1/2_* and V 2*p_3/2_*.


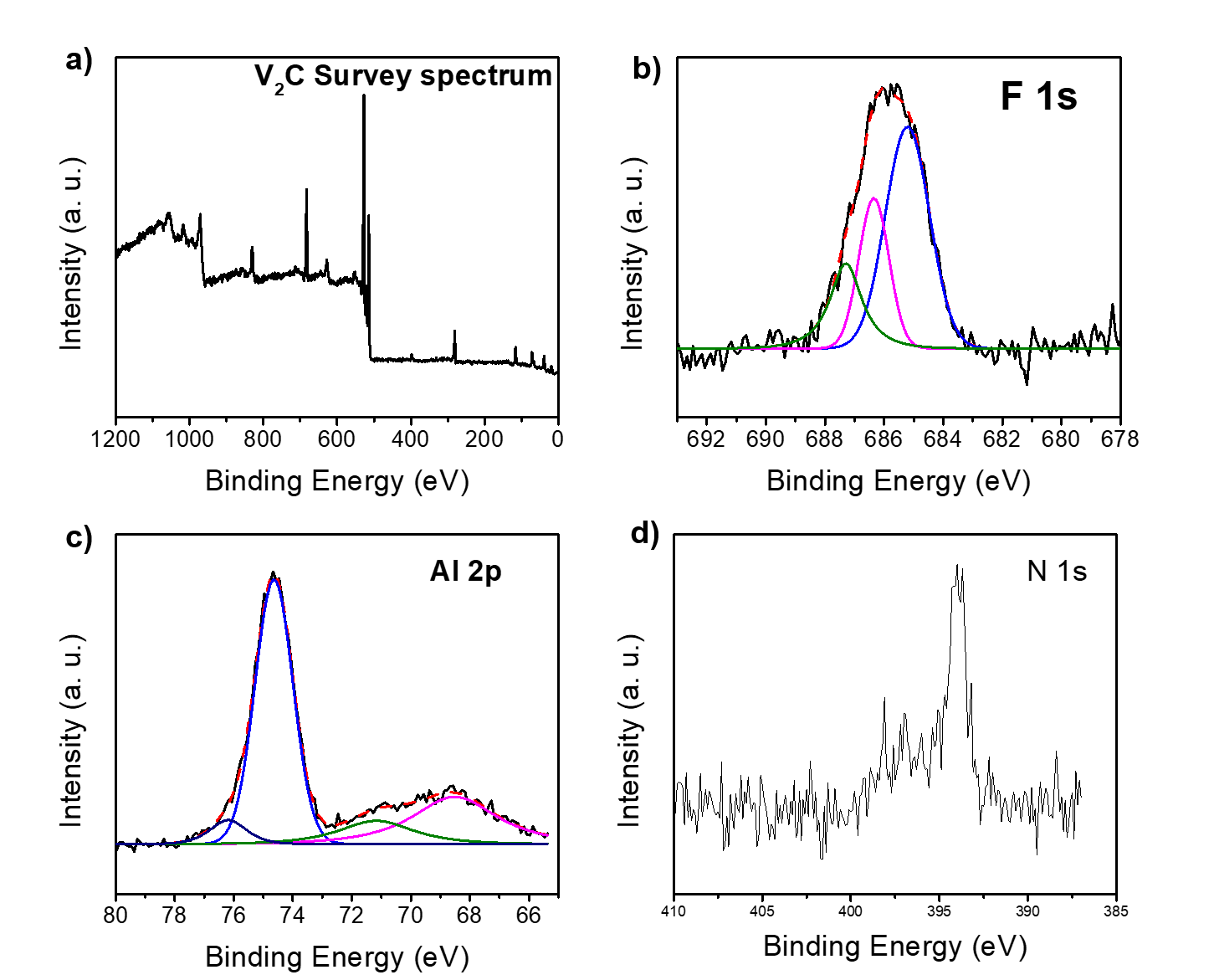


**Fig. S4**. **a)** V_2_CT_x_ survey spectrum and core level spectra of **b)** F 1*s*, **c)** Al 2*p*, and **d)** N 1*s*.

| **Region** | **Assigned to** | **BE (eV)** | **FWHM (eV)** | **Fraction** |
| --- | --- | --- | --- | --- |
| Cr 2p_3/2_ | Cr-C | 573.2 | 1.0 | 0.09 |
|  | Cr-C-O/OH(-H_2_O_ads_) | 574.4 | 1.5 | 0.17 |
|  | Cr(3+)-O (4 peaks) | 575.7-578.5 | 2.0 | 0.69 |
|  | Cr(6+)-O | 579.7 | 2.0 | 0.05 |
| C 1s | C-Cr-T_x_ | 283.0 | 1.1 | 0.01 |
|  | C-C | 284.8 | 1.4 | 0.76 |
|  | C-H/O | 285.8 | 2.2 | 0.16 |
|  | COOH | 288.7 | 1.8 | 0.07 |
| O 1s | Cr-O | 530.0 | 2.0 | 0.15 |
|  | C-Cr-T_x_ | 531.6 | 1.8 | 0.41 |
|  | C-Cr-(OH­)_x_ | 532.5 | 2.0 | 0.27 |
|  | Al(OF)_x_ | 534.8 | 1.9 | 0.17 |
| F 1s | C-Cr-F_x_ | 684.9 | 1.7 | 0.05 |
|  | Cr-F_x_ | 686.2 | 1.6 | 0.85 |
|  | Cr(OF)_x_ | 687.5 | 2.5 | 0.10 |
| Al 2p | Al_2_O­_3_ | 73.5 | 2.6 | 0.06 |
|  | Al-F_x_ | 75.9 | 1.5 | 0.92 |
|  | Al(OF)_x_ | 77.4 | 1.2 | 0.02 |

**Table S5.** Cr_2_CT_x_ XPS peak assignment and peak fitting parameters. The numbers in brackets in the column are the spin-orbit splitting values of Cr 2*p_1/2_* and Cr 2*p_3/2_*.


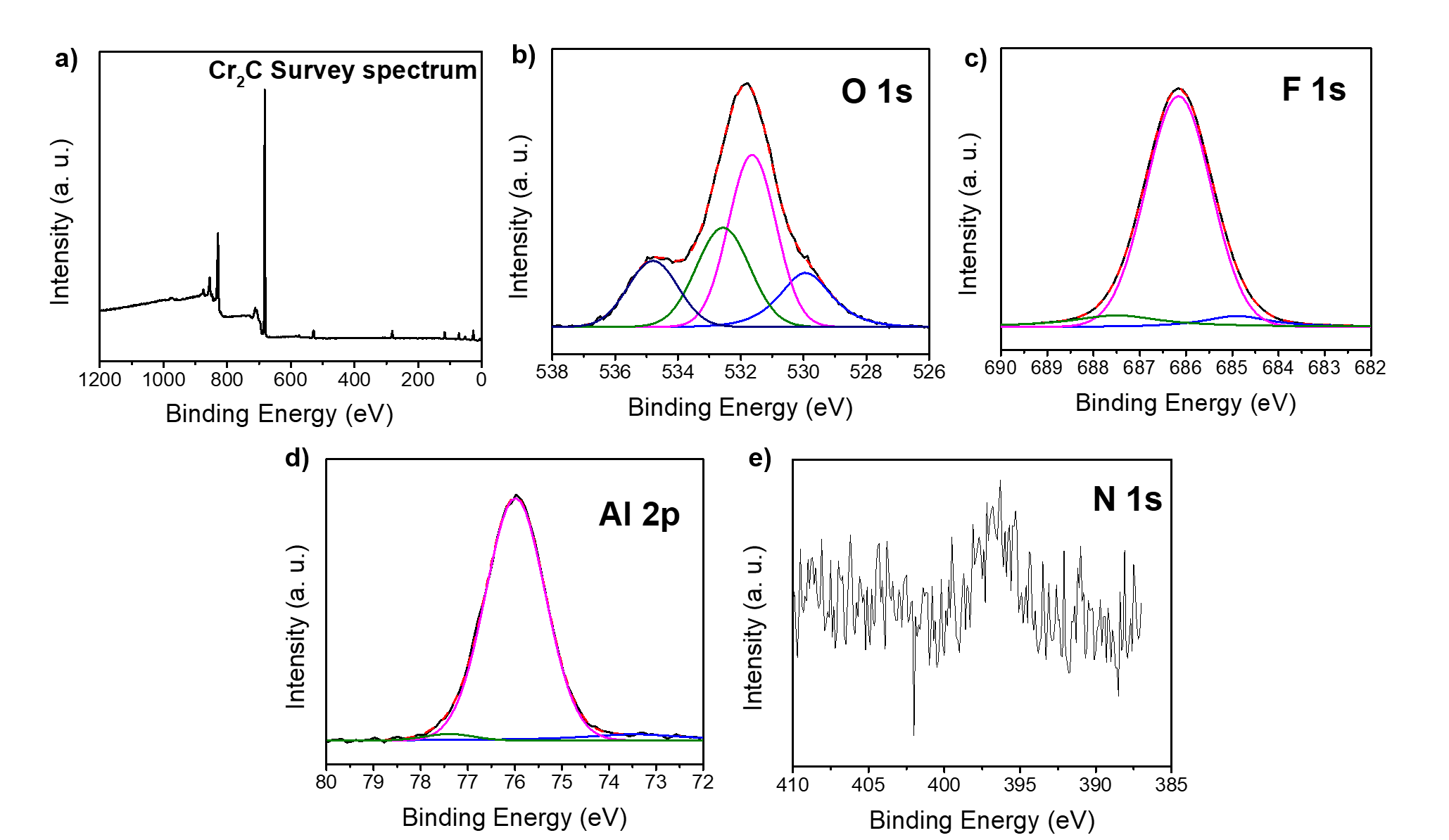


**Fig. S5. a)** Cr_2_CT_x_ survey spectrum and core level spectra of **b)** O 1*s*, **c)** F 1*s*, **d)** Al 2*p*, and **e)** N 1*s*.

| **Region** | **Assigned to** | **BE (eV)** | **FWHM (eV)** | **Fraction** |
| --- | --- | --- | --- | --- |
| Nb 3d_5/2_ | Nb-C | 203.6 (2.8) | 0.9 | 0.03 |
|  | Nb-C-O/OH(-H_2_O_ads_) | 203.8 (2.8) | 0.7 | 0.03 |
|  | Nb(2+)-O | 204.4 (2.8) | 0.8 | 0.02 |
|  | Nb(3+)-O | 205.2 (2.8) | 1.0 | 0.02 |
|  | Nb(4+)-O | 207.0 (2.8) | 0.8 | 0.21 |
|  | Nb(5+)-O | 207.4 (2.8) | 1.1 | 0.57 |
|  | C-Nb-F_x_ | 208.1 (2.8) | 1.1 | 0.12 |
| C 1s | Nb-C | 282.1 | 1.3 | 0.03 |
|  | C-C | 284.8 | 1.5 | 0.71 |
|  | C-H/O | 286.1 | 1.5 | 0.15 |
|  | COO | 288.3 | 2.4 | 0.11 |
| O 1s | Nb-O | 530.5 | 1.2 | 0.31 |
|  | C-Nb-O_x_ | 531.0 | 1.1 | 0.31 |
|  | C-Nb-(OH­)_x_ | 532.0 | 1.5 | 0.19 |
|  | Organic O, H_2_O_ads_ | 533.3 | 1.7 | 0.18 |
|  | Al(OF)_x_ | 534.4 | 0.9 | 0.01 |
| F 1s | Nb-F | 683.2 | 2.0 | 0.02 |
|  | C-Nb-F_x_ | 685.3 | 1.6 | 0.91 |
|  | Al(OF)_x_ | 687.0 | 2.0 | 0.07 |
| Al 2p | Al_2_O­_3_ | 74.2 | 1.4 | 0.08 |
|  | Al-F_x_ | 75.9 | 1.7 | 0.52 |
|  | Al(OF)_x_ | 77.6 | 2.0 | 0.40 |

**Table S6.** Nb_2_CT_x_ XPS peak assignment and peak fitting parameters. The numbers in brackets in the column are the spin-orbit splitting values of Nb 3*d_5/2_* and 2*d_3/2_*.


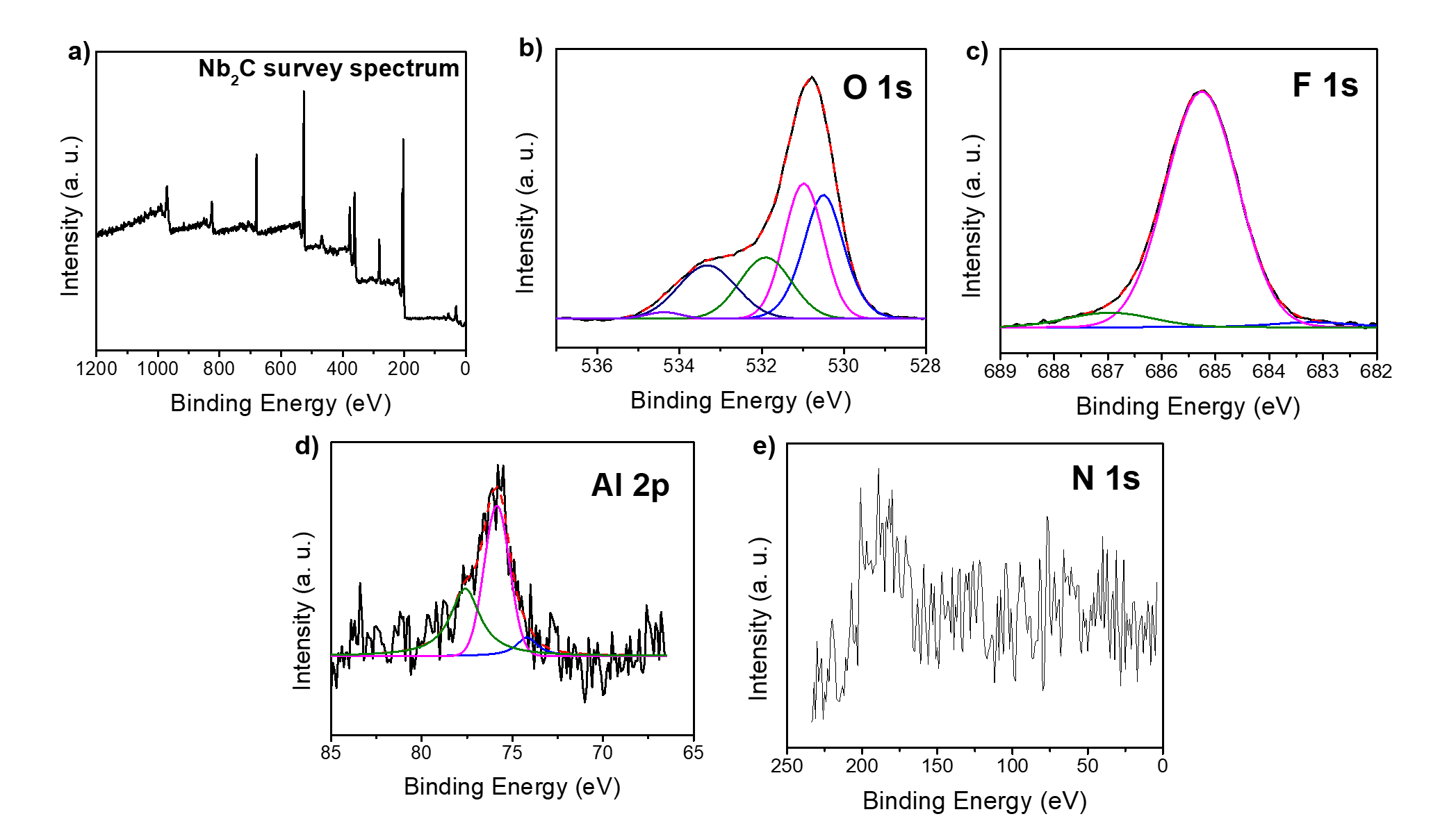


**Fig. S6. a)** Nb_2_CT_x_ survey spectrum and core level spectra of **b)** O 1*s*, **c)** F 1*s*, **d)** Al 2*p*, and **e)** N 1*s*.

| **Region** | **Assigned to** | **BE (eV)** | **FWHM (eV)** | **Fraction** |
| --- | --- | --- | --- | --- |
| Ta 4f_7/2_ | TaC_x_ | 22.2 (1.9) | 0.8 | 0.18 |
|  | Ta^x+^-C-O/OH(-H_2_O_ads_) | 23.0 (1.9) | 1.4 | 0.53 |
|  | TaO_x_ | 26.2 (1.9) | 1.4 | 0.29 |
| C 1s | C-Ta-T_x_ | 282.2 | 1.0 | 0.11 |
|  | C-C | 284.8 | 1.6 | 0.66 |
|  | C-O/H | 286.3 | 1.4 | 0.10 |
|  | C-OOH | 288.8 | 2.7 | 0.12 |
| O 1s | TaO_x_ | 530.0 | 1.4 | 0.32 |
|  | C-Ta-O_x_ | 531.5 | 2.1 | 0.61 |
|  | Organic O, H_2_O_ads_ | 533.0 | 2.0 | 0.07 |
| F 1s | C-Ti-F_x_ | 685.0 | 2.0 | 0.88 |
|  | Al-F_x_ | 685.7 | 1.8 | 0.12 |
| Al 2p | Al-Al | 71.7 | 2.1 | 0.06 |
|  | Al_2_O­_3_ | 74.2 | 1.1 | 0.93 |
|  | Al(OF)_x_ | 77.2 | 0.3 | 0.01 |

**Table S7.** Ta_2_CT_x_ XPS peak assignment and peak fitting parameters. The numbers in brackets in the column are the spin-orbit splitting values of Ta 4*f_7/2_* and 4*f_5/2_*.


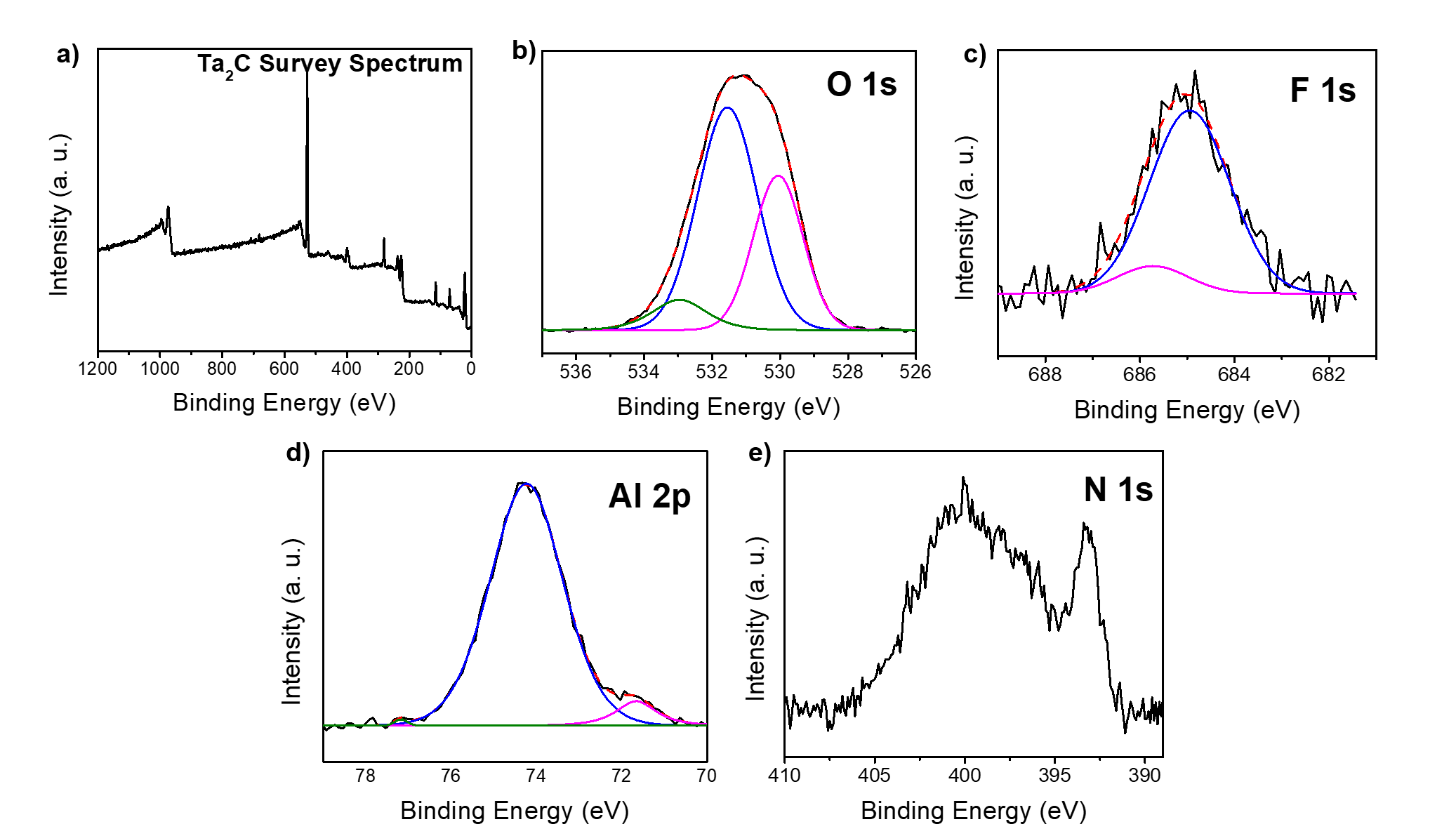


**Fig. S7. a)** Ta_2_CT_x_ survey spectrum and core level spectra of **b)** O 1*s*, **c)** F 1*s*, **d)** Al 2*p*, and **e)** N 1*s*.

**
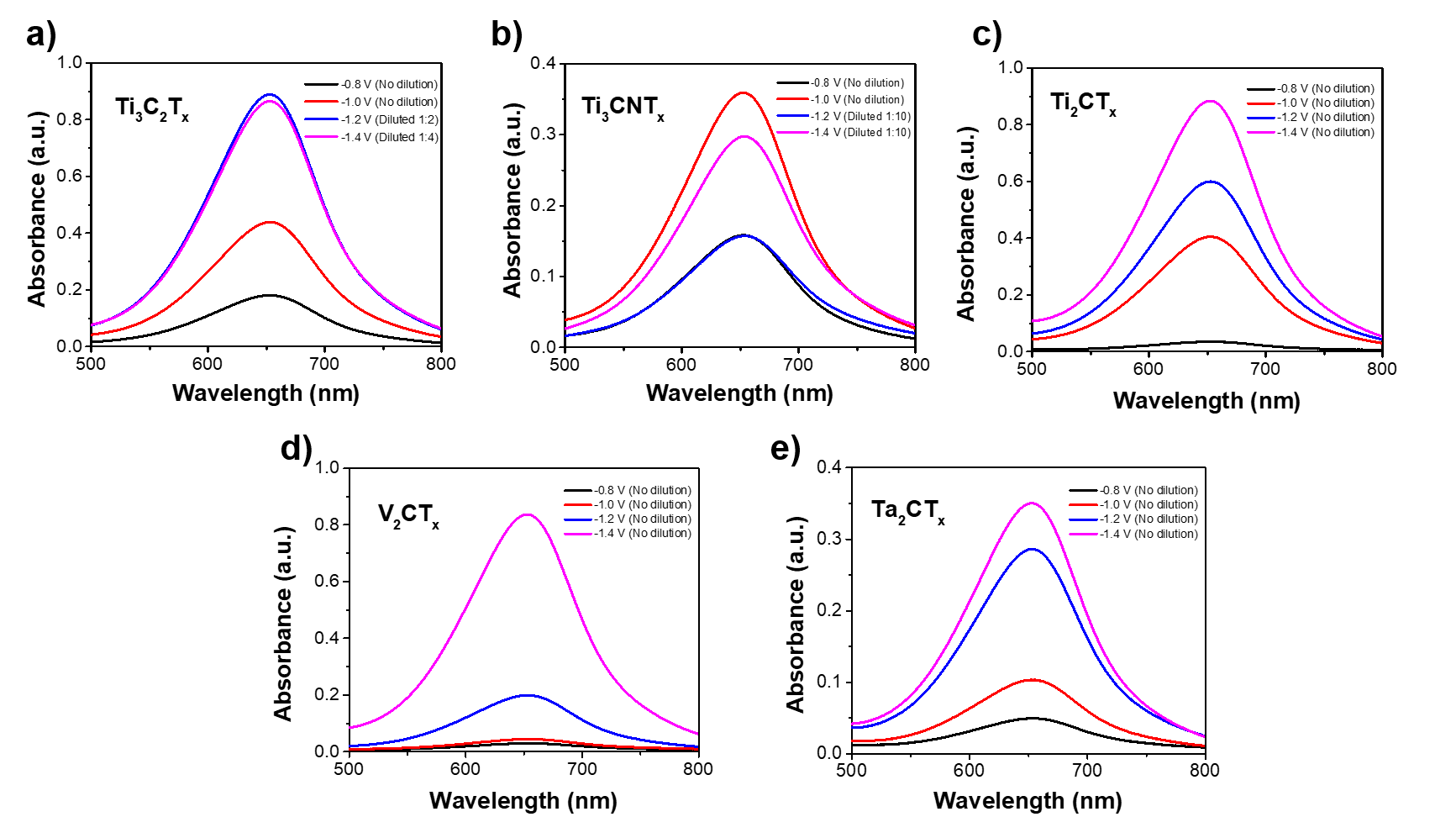
**

**Fig. S8**. UV-Vis absorbance spectra of solutions collected after electrolysis using different MXenes as electrocatalysts. **a)** Ti_3_C_2_T_x_, **b)** Ti_3_CNT_x_, **c)** Ti_2_CT_x_, **d)** V_2_CT_x_, and **e)** Ta_2_CT_x_.


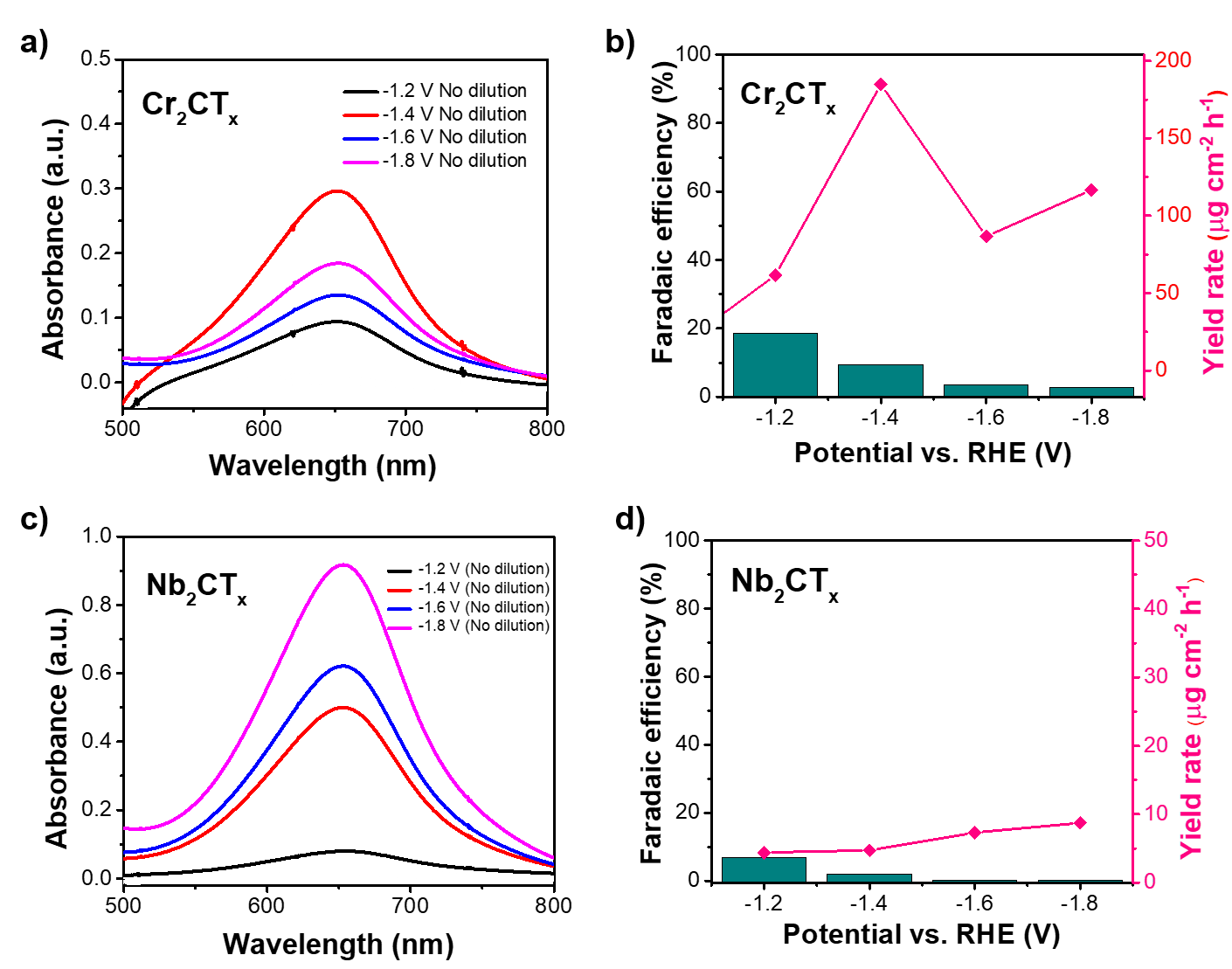


**Fig. S9**. **a)** UV-Vis absorbance spectra of the electrolyte from Nb_2_CT_x_ catalyzed electrochemical conversion of nitrate to ammonia; **b)** faradaic efficiency (FE) and yield rates (YR) of Nb_2_CT_x_. **c)** UV-Vis absorbance spectra of the electrolyte from Cr_2_CT_x_ catalyzed conversion and **d)** FE and YR Cr_2_CT_x_.

**
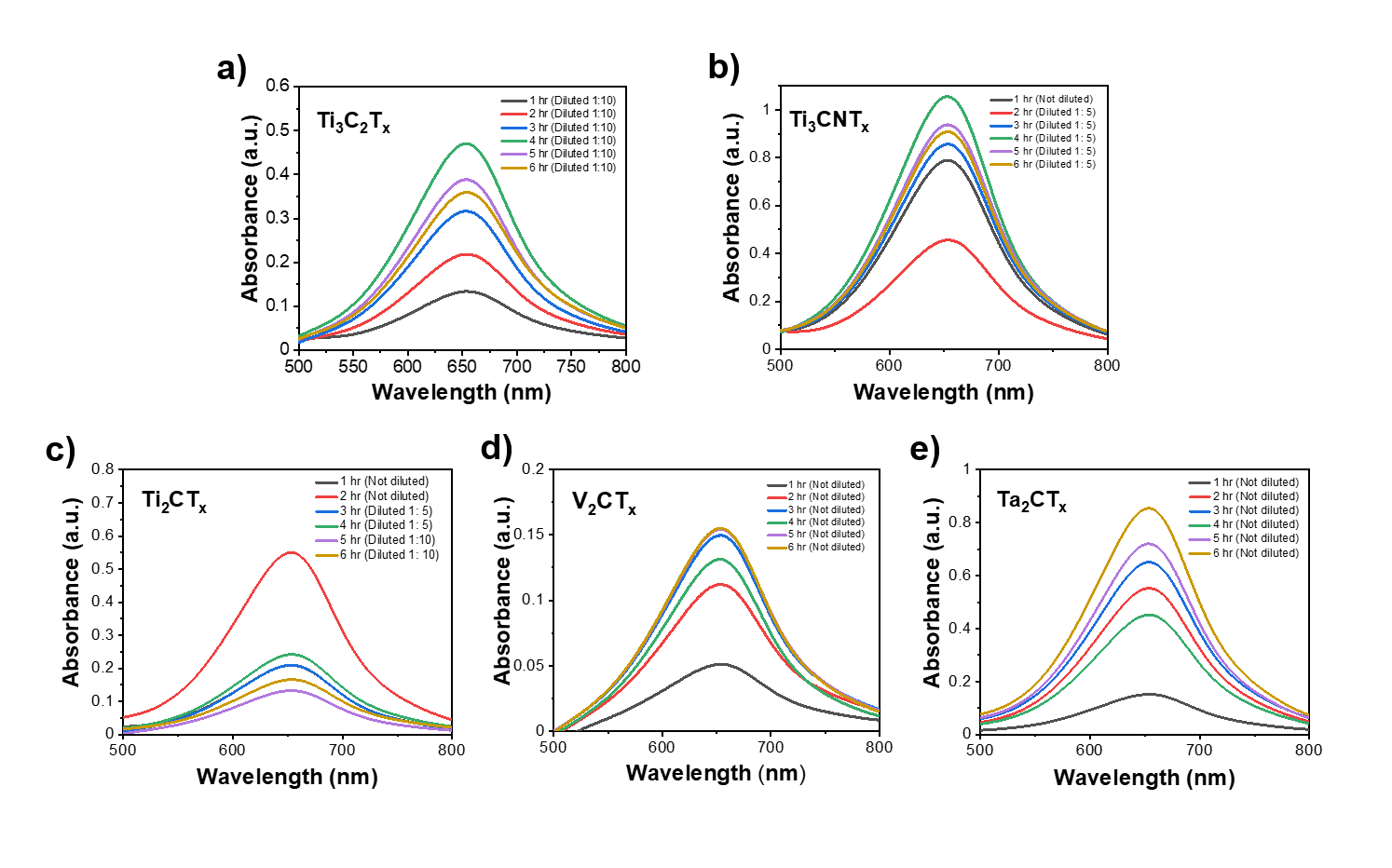
**

**Fig. S10**. UV-Vis absorbance spectra with appropriate dilution for stability test from electrochemical nitrate reduction conversion to ammonia based on **a)** Ti_3_C_2_T_x_, **b)** Ti_3_CNT_x_, **c)** Ti_2_CT_x_, **d)** V_2_CT_x_, and **e)** Ta_2_CT_x_.

**
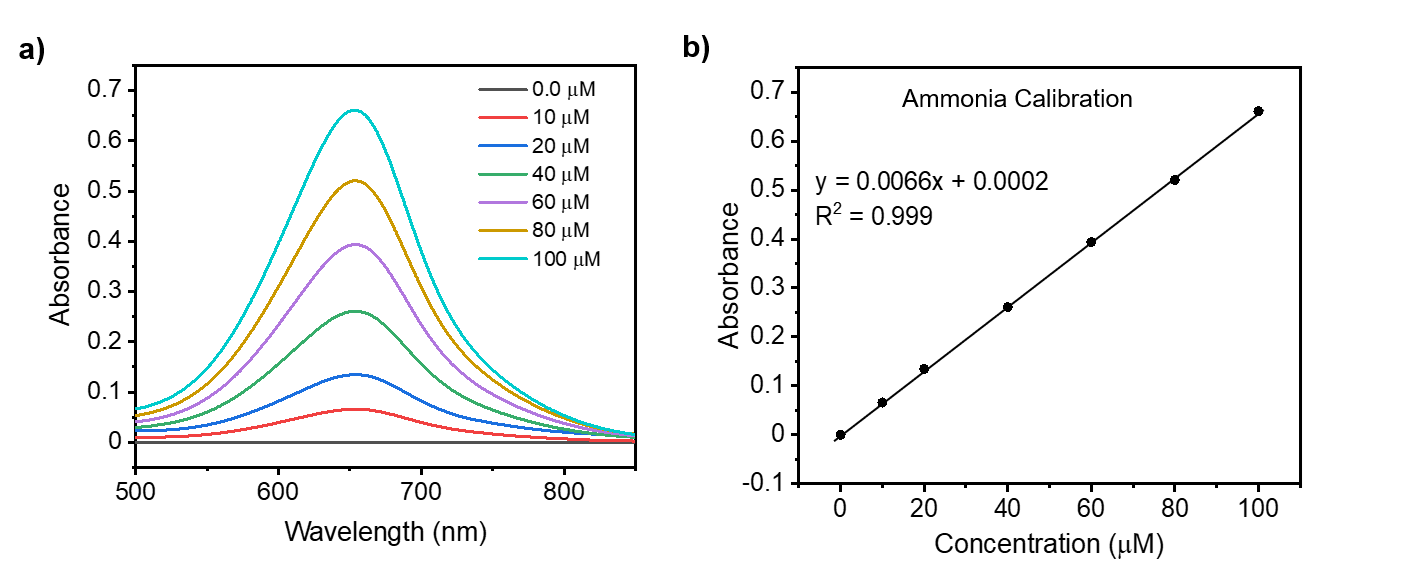
**

**Fig. S11. a)** UV-Vis spectra obtained in the indophenol blue colorimetric method for ammonia determination. The absorbance of the spectra at each concentration was measured at 655 nm. **b)** Calibration curve obtained by plotting the absorbance at 655 nm at each concentration point of NH_4_Cl.


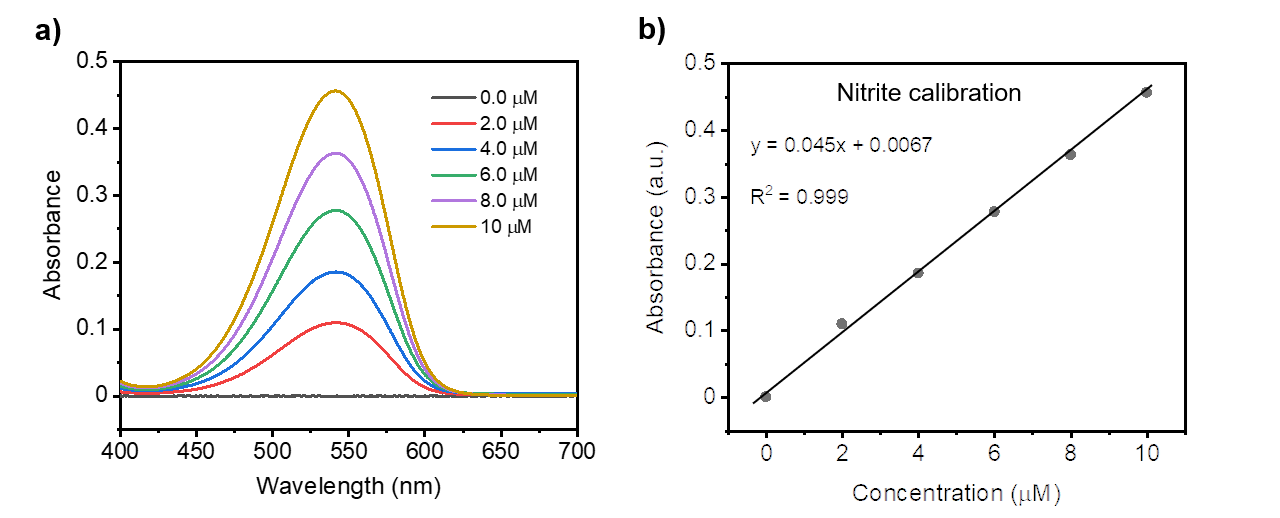


**Fig S12. a)** UV-Vis spectra obtained in the colorimetric method for nitrite determination. Absorbance of the spectra at each concentration was measured at 540 nm. **b)** Calibration curve obtained by plotting the absorbance at 540 nm at each concentration point of NaNO_2_.

**
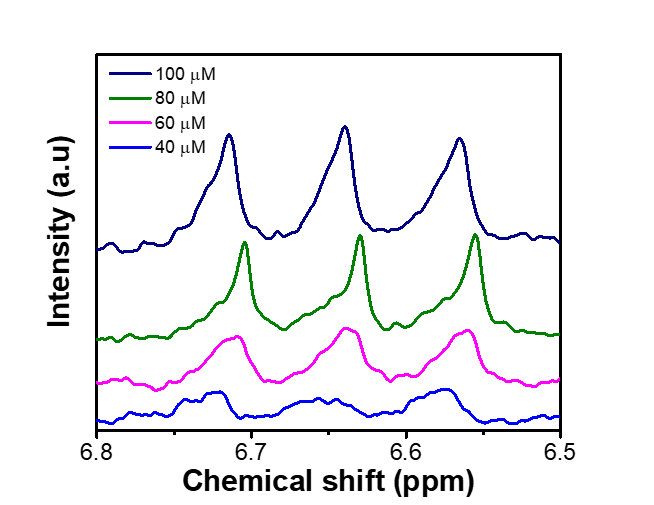
**

**Figure S13.** Spectra obtained in the ammonia determination analyzed by 1H-NMR quantitative method. Spectra correspond to a normalization by the area of the internal standard (maleic acid). Calibration curve was carried out using the area under the complete signal corresponding to NH_4_^+^. Standards were prepared using NH_4_Cl.

**
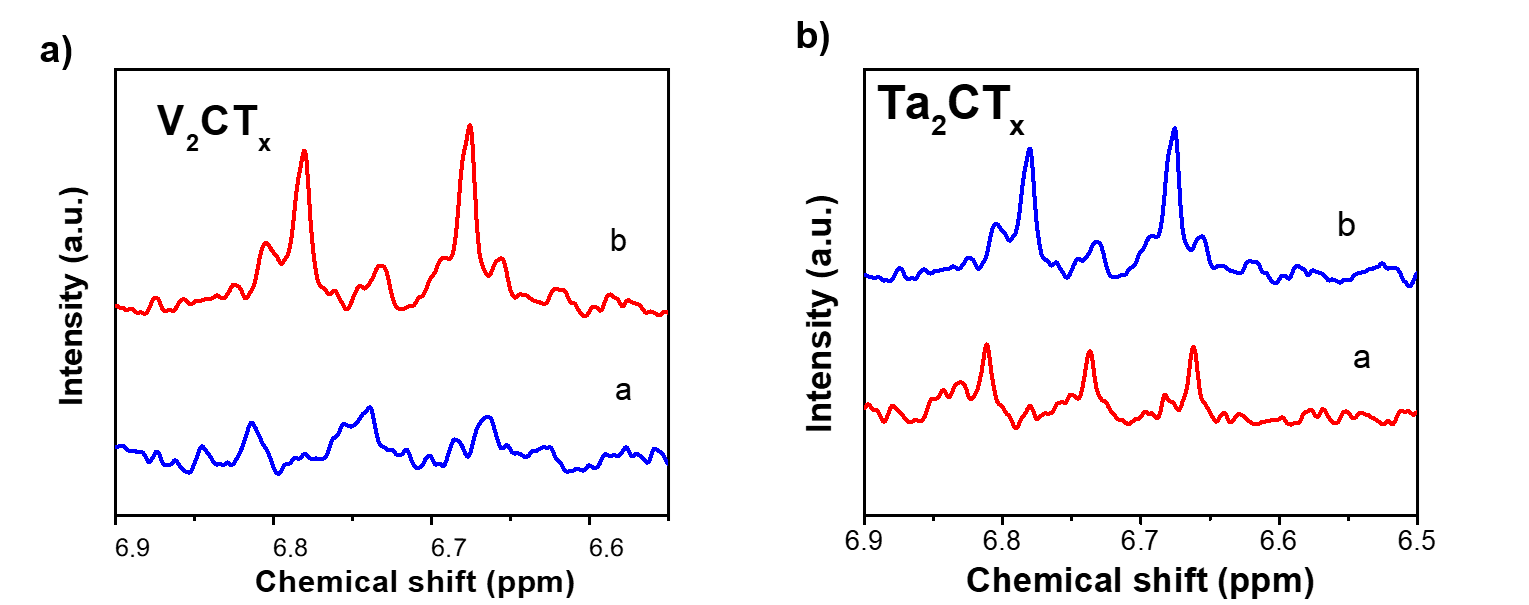
**

**Fig S14.** ^1^H NMR spectra of the electrolyte after electrocatalytic nitrate-to-ammonia conversion at −1.2 V, using K^14^NO_3_ (a) and K^15^NO_3_ (b) as the nitrogen source, catalyzed by **a)** V_2_CT_x_ **b)** Ta_2_CT_x_


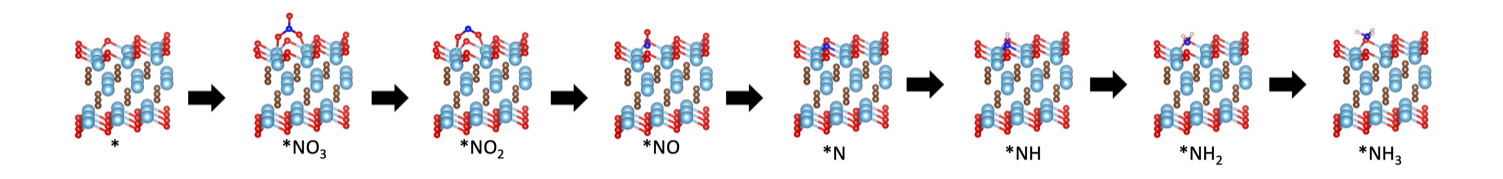
**Fig. S15.** Optimized structures of NO_3_RR intermediates adsorbed on O_v_-Ti_3_C_2_ MXene. Coloring: Ti-sky blue, O-red, N-dark blue, C-brown, H-white.


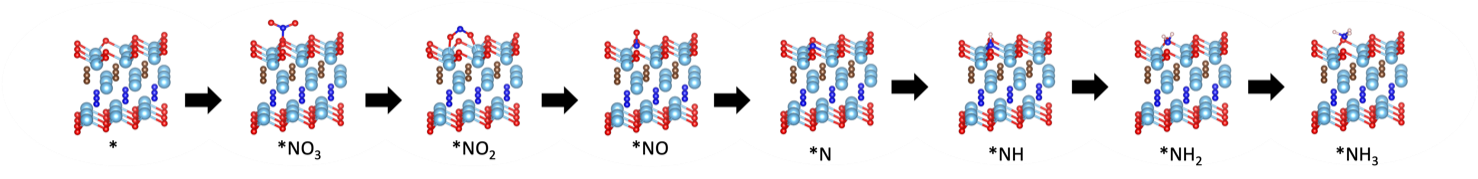


**Fig. S16.** Optimized structures of NO_3_RR intermediates adsorbed on O_v_-Ti_3_CN MXene. Coloring: Ti-sky blue, O-red, N-dark blue, C-brown, H-white.


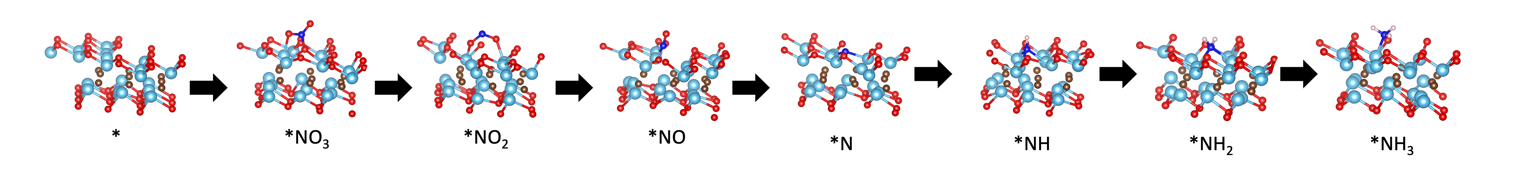


**Fig. S17.** Optimized structures of NO_3_RR intermediates adsorbed on O_v_-Cr_2_C MXene. Coloring: Cr-sky blue, O-red, N-dark blue, C-brown, H-white.


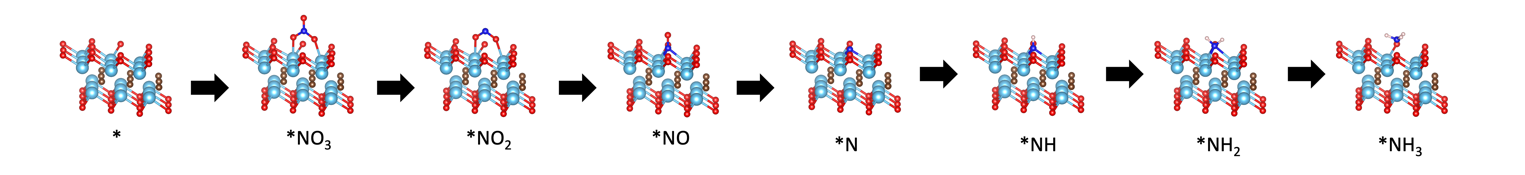


**Fig. S18.** Optimized structures of NO_3_RR intermediates adsorbed on O_v_-Nb_2_C MXene. Coloring: Nb-sky blue, O-red, N-dark blue, C-brown, H-white.


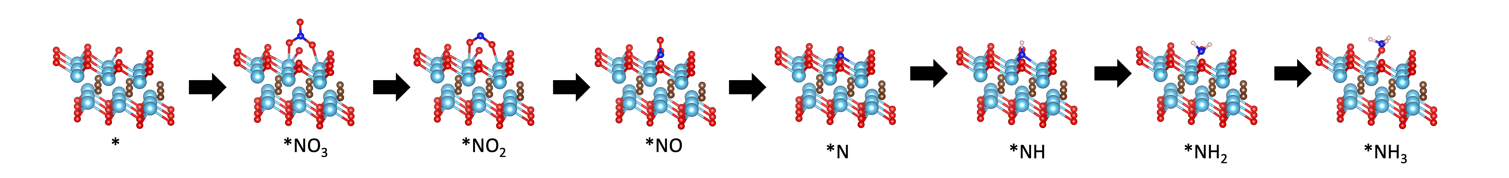


**Fig. S19.** Optimized structures of NO_3_RR intermediates adsorbed on O_v_-Ta_2_C MXene. Coloring: Ta-sky blue, O-red, N-dark blue, C-brown, H-white.


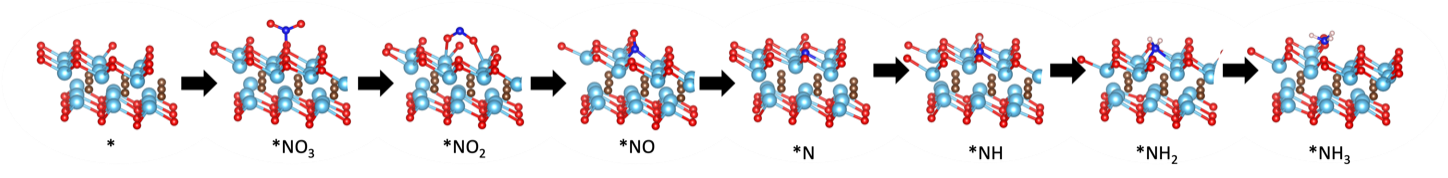


**Fig. S20.** Optimized structures of NO_3_RR intermediates adsorbed on O_v_-V_2_C MXene. Coloring: V-sky blue, O-red, N-dark blue, C-brown, H-white.


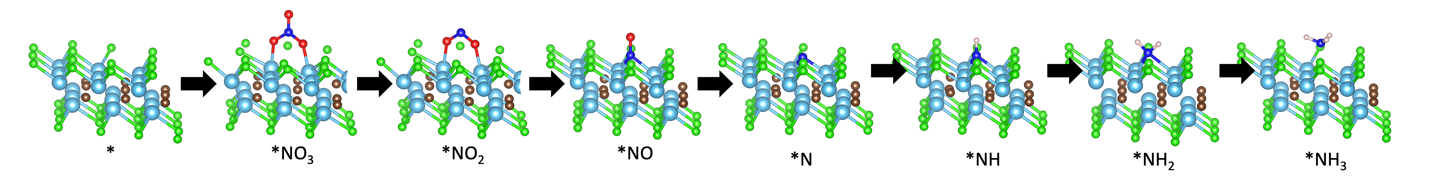


**Fig. S21.** Optimized structures of NO_3_RR intermediates adsorbed on F_v_-Cr_2_C MXene. Coloring: Cr-sky blue, O-red, N-dark blue, C-brown, H-white, F-green.


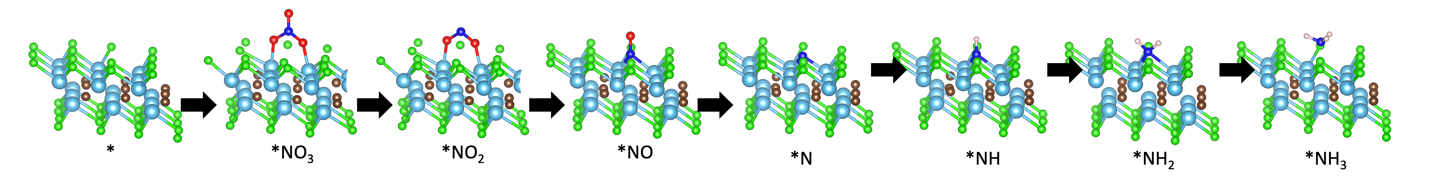


**Fig. S22.** Optimized structures of NO_3_RR intermediates adsorbed on F_v_-Nb_2_C MXene. Coloring: Nb-sky blue, O-red, N-dark blue, C-brown, H-white, F-green.


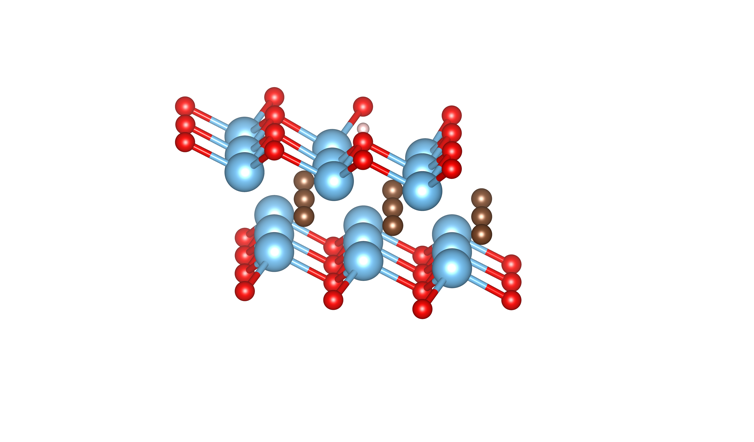


**Fig. S23.** Representative example of the adsorption configuration of *H on O_v_-Ti_2_C MXene. Coloring: Ti-sky blue, O-red, N-dark blue, C-brown, H-white.

REFERENCES

(1) Halim, J.; Cook, K. M.; Naguib, M.; Eklund, P.; Gogotsi, Y.; Rosen, J.; Barsoum, M. W. X-Ray Photoelectron Spectroscopy of Select Multi-Layered Transition Metal Carbides (MXenes). *Applied Surface Science* 2016, *362*, 406–417. https://doi.org/10.1016/j.apsusc.2015.11.089.
